# Sporulation conditions influence the surface and adhesion properties of *Bacillus subtilis* spores

**DOI:** 10.3389/fmicb.2023.1219581

**Published:** 2023-09-01

**Authors:** Audrey Hamiot, Christelle Lemy, Frederic Krzewinski, Christine Faille, Thomas Dubois

**Affiliations:** ^1^Univ. Lille, CNRS, INRAE, Centrale Lille, UMR 8207 - UMET - Unité Matériaux et Transformations, Lille, France; ^2^Univ. Lille, CNRS, UMR 8576 - UGSF - Unité de Glycobiologie Structurale et Fonctionnelle, Lille, France

**Keywords:** *Bacillus subtilis*, sporulation, spores, crust, adhesion, hydrophobicity, glycans

## Abstract

Spore-forming bacteria of the *Bacillus subtilis* group are responsible for recurrent contamination of processing lines in the food industry which can lead to food spoilage. The persistence of *B. subtilis* would be due to the high resistance of spores to extreme environmental condition and their propensity to contaminate surfaces. While it is well known that sporulation conditions modulate spore resistance properties, little is known about their effect on surface and adhesion properties. Here, we studied the impact of 13 sporulation conditions on the surface and adhesion properties of *B. subtilis* 168 spores. We showed that Ca^2+^ or Mg^2+^ depletion, lower oxygen availability, acidic pH as well as oxidative stresses during sporulation lead to the release of more hydrophobic and adherent spores. The consequences of these sporulation conditions on crust composition in carbohydrates and proteins were also evaluated. The crust glycans of spores produced in a sporulation medium depleted in Ca^2+^ or Mg^2+^ or oxygen-limited conditions were impaired and contained lower amounts of rhamnose and legionaminic acid. In addition, we showed that lower oxygen availability or addition of hydrogen peroxide during sporulation decreases the relative amount of two crust proteins (CgeA and CotY) and the changes observed in these conditions could be due to transcriptional repression of genes involved in crust synthesis in late stationary phase. The fact that sporulation conditions affect the ease with which spores can contaminate surfaces could explain the frequent and recurrent presence of *B. subtilis* spores in food processing lines.

## 1. Introduction

Spore-forming bacteria of the *Bacillus subtilis* group, including *B. subtilis sensu stricto*, *B. amyloliquefaciens*, and *B. licheniformis*, are a major economic concern in the food industry. Indeed, these bacteria are among the most commonly identified species in spoiled heat-treated food. For example, *B. amyloliquefaciens* is commonly responsible for the contamination of bakery products, causing notably ropey bread spoilage while *B. licheniformis* is often involved in the contamination of dairy products (André et al., 2017). The raw materials (food, ingredients) but also the surfaces of the food processing lines would be the major sources of contamination by these spore-forming bacteria. *Bacillus* spores are known to be highly adherent to all types of surfaces and resistant to most transformation and hygiene procedures encountered in food processing plants (André et al., 2017). Therefore, after cleaning and disinfection procedures, viable adherent *Bacillus* spores are still commonly found on equipment surfaces and thus potentially initiate new contaminations in subsequent processing cycles by germinating, multiplying, and then forming biofilms in which sporulation is often observed (Soni et al., 2016).

*Bacillus subtilis* spores are oval-shaped structures formed by concentric layers surrounding the core (McKenney et al., 2013; Driks and Eichenberger, 2016) that contain the chromosomal DNA wrapped around Small Acid Soluble Proteins. The core is surrounded, from innermost to outermost, by the inner membrane, the germ cell wall, the cortex, the outer spore membrane, and the coat. The coat contains more than 80 proteins and represents around 25% of total spore proteins. The coat is subdivided into three parts, the basement layer, the inner coat, and the outer coat. The coat assembly is guided by five morphogenetic proteins (SpoIVA, SafA, CotE, SpoIVM, and SpoVID) that play a key role in coat morphogenesis. Most of the spores of strains of the *B. subtilis* group are also surrounded by an outermost layer, called the crust (Zhang et al., 1994; McKenney et al., 2010; Faille et al., 2014). Faille and colleagues removed the crust of *B. subtilis* spores by a mechanical treatment with a French press. The removal of the crust changed these spores from hydrophilic to hydrophobic and increased their adhesion to stainless steel demonstrating that the crust gives *B. subtilis* spores their specific surface properties, notably a marked hydrophilic character. It was deduced that the crust could be the reason for the relatively low capacity of *B. subtilis* spores to contaminate surfaces, compared to other *Bacillus* species such as *B. cereus* (Faille et al., 2014).

The crust is composed of proteins and carbohydrates (Faille et al., 2014; Bartels et al., 2019; Shuster et al., 2019a,b; Dubois et al., 2020). So far, six proteins have been identified in the crust: CotV, CotW, CotX, CotY, CotZ, and CgeA (Bartels et al., 2019; Shuster et al., 2019a). CotX, CotY, and CotZ are the morphogenetic proteins of the crust and CotZ could be the major one, as the localization of all crust proteins, except CotW, depends on it (Bartels et al., 2019; Shuster et al., 2019a). These proteins are rich in cysteine residues and form an insoluble protein fraction suggesting the presence of disulfide cross-link between these proteins (Ursem et al., 2021). The protein backbone of the crust is predominantly formed by CotY and to a less extent by CotV and CotX (Bartels et al., 2019). When overexpressed in *Escherichia coli*, CotY self-assembles into crystal structures that tend to form multilayered stacks (Jiang et al., 2015). How the crust is anchored to the spore surface remains to be investigated. However, it was suggested that CotW is a linker protein at the interface of the outer coat and the crust (Bartels et al., 2019). Accordingly, it was shown by transmission electron microscopy that the crust of a ∆*cotW* mutant is disorganized and separated from the coat (Shuster et al., 2019a). In a heterologous host, CotW and CotV were self-organized into fibrous assemblies of consistent diameter (Jiang et al., 2015). Therefore, it is likely that the fibrous assembly formed by the CotV-CotW complex participates in the anchoring of the crust to the spore surface. Finally, the function of CgeA is still to be determined but CgeA might be a target of glycosylation or a glycosylation hub playing a role in coordinating the glycosylation of crust proteins (Bartels et al., 2019; Shuster et al., 2019a). Carbohydrates are the other key components of the crust. The crust contains rhamnose (Rha), quinovose (Qui), glucose (Glc), glucosamine (GlcN), and muramic lactam which would play a significant role in spore surface properties since mutations in genes expressed in late sporulation and encoding putative glycosyltransferase modify crust structure and/or spore surface hydrophilicity (Roels and Losick, 1995; Cangiano et al., 2014; Faille et al., 2014; Shuster et al., 2019b). Finally, the spore surface of *B. subtilis* is covered with legionaminic acid (Leg), a nine-carbon backbone nonulosonic acid, whose biosynthesis pathway is required for proper crust assembly (Dubois et al., 2020). Nevertheless, the structure and localization of crust carbohydrates remain poorly described.

Previous studies demonstrated that environmental conditions during sporulation can modulate spore resistance properties (Bressuire-Isoard et al., 2018). For example, it has been shown that supplementation of the sporulation medium with divalent cations such as Ca^2+^, Mg^2+^, or Fe^2+^ increases the resistance to wet heat of *B. subtilis* spores while the addition of some amino acids such as thioproline or cysteine improves *B. subtilis* spore resistance to UV-A, UV-B and peracetic acid (Cazemier et al., 2001; Moeller et al., 2011). Other parameters such as pH and temperature during sporulation can also affect spore resistance to wet heat or chemical denaturants (Melly et al., 2002; Baweja et al., 2008). In *B. anthracis*, spores produced at pH 5 are more resistant to wet heat, and sporulation at pH 9 leads to spores more resistant to sodium hydroxide, compared to spores produced at pH 7 (Baweja et al., 2008). Lastly, *B. subtilis* produced spores that are more resistant to wet heat when sporulation occurs at 37°C rather than 30°C (Melly et al., 2002). As illustrated by these few examples, most of the works on sporulation conditions have focused on spore resistance, while little is known about the effects of sporulation conditions on the surface and adhesion properties of spores. On *B. subtilis* spores, a study conducted in 1990 showed minor changes in the hydrophobic character of spores produced in different culture media (Wiencek et al., 1990). More recently, studies on *B. subtilis* spores have shown that pH and temperature during sporulation affect spore surface properties. Indeed, *B. subtilis* spores produced at 25°C are more hydrophobic than those produced at 37°C (Isticato et al., 2020). The hydrophobic character of the *B. subtilis* spores is also affected by the pH of the sporulation medium since it increases with pH for values between 7.0 and 8.5 (Eschlbeck et al., 2017).

In this study, we investigated the influence of environmental conditions encountered by *B. subtilis* 168 during sporulation on spore surface and adhesion properties. For sporulation conditions leading to an increase in spore adhesion, we investigated the crust structure and composition in carbohydrates and proteins to identify the changes responsible for the increased adhesion. Finally, we performed qRT-PCR experiments to determine whether the changes in crust composition can be explained by transcriptional regulation of genes involved in crust synthesis during the late sporulation phase.

## 2. Materials and methods

### 2.1. Bacterial strains and growth conditions

Sporulation experiment were performed with the *B. subtilis* 168 strain (BGSC, 1A1). *Escherichia coli* K-12 strain TG1 was used as a host for the construction of plasmids and cloning experiments. *E. coli* strains were grown in Lysogeny Broth (LB, Sigma-Aldrich, L3022) at 37°C. *Bacillus* strains were grown at 30 or 37°C in LB, Nutrient Broth (NB, Biokar, BK003HA) or Spo8, a sporulation medium composed of 12.3 g/L NB, 0.51 g/L MgSO_4_ 7H_2_O, 0.97 g/L KCl, 0.2 g/L CaCl_2_ 2H_2_O, 3 mg/L MnCl_2_ 4H_2_O and 0.55 mg/L FeSO_4_ 7H_2_O at pH 6.9 (Faille et al., 2013). The following concentrations of antibiotic were used for bacterial selection: 100 μg/mL ampicillin or 20 μg/mL chloramphenicol for *E. coli* and 5 μg/mL chloramphenicol for *Bacillus*.

For sporulation experiments in the Spo8 condition (control), the following procedure was achieved. Sterile Spo8 medium was inoculated at an optical density at 600 nm of 0.05 with an overnight preculture of the *B. subtilis* 168 strain. Then, the culture was incubated at 30°C under agitation (220 rpm). After 5 days, spores were harvested by centrifugation (2500 *g* for 15 min, 4°C), washed three times in chilled sterile water, and stored at 4°C. After 1 week, two additional washes were performed to remove the residual vegetative cell debris and spores were stored at 4°C in sterile water until use for up to 3 months. Spores were checked under microscope and spore preparations with more than 98% of spores were kept for further experiments. The other sporulation conditions are listed in Table 1. Most of the changes in sporulation conditions (addition of chemicals, UV treatment) were implemented 2 h after the transition to the stationary phase. Only the sporulation phase was therefore affected. The chemicals used are listed in Supplementary Table 1. UV treatment was performed by exposing the culture to 254 nm UV radiations (Kingrate, UVC 6WT5, China) for 30 min while maintaining agitation and temperature. In -CaCl_2_ and -MgSO_4_ conditions, Spo8 medium depleted in CaCl_2_ or MgSO_4_ was inoculated and incubated as described above. Therefore, the bacteria were subjected to these depletions from the beginning of the growth. Ca^2+^ and Mg^2+^ concentrations in Spo8 and Spo8 media depleted in CaCl_2_ or MgSO_4_ were determined using the Varian SpectrAA-55B atomic absorption spectrophotometer with a Varian SpectrAA Ca/Mg lamp. A standard range was used for each cation. Ca^2+^ was detected at *λ* = 422.7 nm and Mg^2+^ at *λ* = 285.2 nm. Before analysis, the three Spo8 growth media were diluted in a solution of lanthanum (III) chloride hexahydrate (10 g/L) and hydrochloric acid (84 mL/L of 37% solution).

**Table 1:** Sporulation conditions.

Sporulation condition	Description	Consequence
Spo8	Control	–
**Modifications of Spo8 from the beginning of the growth**		
-CaCl_2_	Spo8 without CaCl_2_ complementation	Depletion in Ca^2+^ (Supplementary Figure 1A)
-MgSO_4_	Spo8 without MgSO_4_ complementation	Depletion in Mg^2+^ (Supplementary Figure 1B)
**Modifications of Spo8 during the stationary phase**		
-O_2_	The Erlenmeyer containing the bacterial culture was sealed with a rubber stopper	Modified atmosphere (progressive decrease in the O_2_ availability)
-O_2_ + CO_2_	-O_2_ condition with the addition of 0.8% sodium bicarbonate	Modified atmosphere (progressive decrease in the O_2_ availability and increased CO_2_ concentration) and increase of pH (Supplementary Figure 1C)
H_2_O_2_	Addition of 0.005% of H_2_O_2_	Oxidative stress
Peracetic acid	Addition of 0.005% of peracetic acid	Oxidative stress and decrease of pH (Supplementary Figure 1C)
pH 5	Acidification to pH 5 by the addition of HCl	pH decrease
pH 9	Alkalinization to pH 9 by the addition of NaOH	pH increase
Benzoic acid	Addition of 0.05% C₆H₅CO₂H, a food preservative (E210)	Antibacterial properties and decrease of pH (Supplementary Figure 1C)
Nitrites	Addition of 0.05% NaNO_2_, a food preservative (E250)	Antibacterial properties
RBS	Addition of 0.05% RBS 25, an alkaline detergent	Detergent effect without significant impact on pH (Supplementary Figure 1C)
BAC	Addition of 0.00025% Benzalkonium chloride, a quaternary ammonium disinfectant	Antibacterial properties
UV	Ultraviolet treatment at 254 nm	Antibacterial properties

This table summarizes the different sporulation conditions used in the study. Modifications of the sporulation conditions are made from the beginning of the growth or in the early stationary phase (2 h after the transition to the stationary phase). The concentrations in peracetic acid, H_2_O_2_, benzoic acid, nitrites, RBS, and BAC used in these experiments correspond to the maximum concentrations of each of these agents that did not decrease the total number of CFU after 5 days of growth.

### 2.2. Construction of recombinant strains

The plasmids pBS1CΩ*amyE*::*cgeA*-GFP and pBS1CΩ*amyE::cotY*-GFP were constructed as follows. The *gfp*-λter DNA fragment containing the *gfp* gene and the terminator of the λ prophage was obtained by SOE-PCR using the primers gfp-1 / gfp-2 and λter-1 / λter-2. The plasmids pSB1C3-mGFPmut1-orginal and pMUTIN2 were respectively, used as templates (Vagner et al., 1998; Popp et al., 2017). Then, the *cgeA* or *cotY* genes with their respective promoters and *gfp*-λter fragment were amplified by SOE-PCR using primers cgeA-1 / cgeA-2 or cotY-1 / cotY-2 and gfp-1 / λter-2 (Supplementary Table 2). The *B. subtilis* 168 chromosomal DNA and the *gfp*-λter fragment were respectively, used as templates. The latter amplification also introduced a sequence encoding 5 glycines that act as a linker between CotY or CgeA and the GFP. The resulting fragments (*cgeA*-*gfp*-λter and *cotY*-*gfp*-λter) were purified as *Xba*I*-Pst*I fragments and were inserted between the *Xba*I and *Pst*I sites of pBS1C (Radeck et al., 2013). The nucleotide sequences of the fragments inserted into pBS1C were verified by Sanger sequencing (Eurofins Genomics, Germany). *B. subtilis* 168 was then transformed with the plasmids pBS1CΩ*amyE:: cgeA*-GFP or pBS1CΩ*amyE:: cotY*-GFP and homologous recombination at the *amyE* locus was performed as described previously (Radeck et al., 2013). Nucleotide sequences of the introduced chromosomal modifications were verified by Sanger sequencing (Eurofins Genomics, Germany). The *B. subtilis* 168 pHT304-18 P*_sspA_*-*sspA*-GFP strain was obtained by transforming the *B. subtilis* 168 strain with the plasmid pHT304-18 P*_sspA_*-*sspA*-GFP (Richard et al., 2020).

### 2.3. Heat resistance and germination of spores

The percentage of heat-resistant spores was defined as the ratio of the number of colony-forming units (CFU) after heating to the number of CFU before heating. The number of CFU before heating was evaluated after plating the spore suspensions on NB-agar and incubation for 24 h at 37°C. The number of CFU after heating was evaluated by the same procedure after heat treatment of the spore suspensions for 12 min at 80°C. The ability of spores to germinate was assessed by monitoring the decrease in optical density at 580 nm (OD_580_) of spore suspensions which reflects the transition of spores from phase-bright to phase-dark during germination (Xing and Harper, 2020). Spores were heat activated at 70°C for 30 min and chilled on ice for 5 min. Then, the spore suspension was adjusted to an initial OD_580_ of 1 in a 10 mM L-alanine solution in Tris buffer (Tris base 10 mM, NaCl 10 mM, pH 7.4) and the OD_580_ was monitored with a microplate reader (Biotek, synergy HTX) for 2 h at 30°C with a measurement every 2 min following 5 s of stirring.

### 2.4. Adhesion, surface properties, and structure of spores

The adhesion of spores to polypropylene was measured as described previously with the following modifications (Abe et al., 2014). The spore suspension was adjusted to an OD_600_ of about 0.6 in physiological water and 1 mL of the spore suspension was added to a polypropylene tube (2.0 mL Safe-lock tube, Eppendorf). The spore suspension was mixed with a vortex for 10 s to promote spore adhesion to the tube wall, then transferred to a new polypropylene tube. This step was repeated 9 times. The OD_600_ of the spore suspension was measured after 2, 5, 8, and 10 adhesion steps. The adhesion percentage was calculated by following the formula: 100 × [OD_i_ – OD_n_]/OD_i_, where OD_i_ is the initial OD_600_ and OD_n_ is the OD_600_ after n steps.

The hydrophilic/hydrophobic property of spores was characterized by MATH, a partitioning method based on the affinity of spores to the apolar solvent hexadecane (Sigma-Aldrich, H6703), as described previously (Faille et al., 2019). The higher the affinity of the spores for hexadecane, the more hydrophobic the spores. Quickly, spores were suspended in physiological water at an OD_600_ of 0.6 (A0). Three-milliliter aliquots of the spore suspension and 500 μL of hexadecane were placed in glass tubes (10 mm × 75 mm), vortexed for times ranging from 5 s to 240 s, and left to settle for 30 min to allow the two phases to separate. The OD_600_ of the aqueous phase was then measured (At). Aeq is the asymptotic or lowest absorbance value obtained. The Gibbs energy of partitioning (ΔGpar) is obtained from the equilibrium constant *K* (ΔGpar = log K), which expresses the partitioning of bacteria between the aqueous and hexadecane phases. It was calculated from the Equation *K* = [6(A0–Aeq)/Aeq].

To measure the global charge of the spore surface, zetametry experiments were performed as previously described (Faille et al., 2014).

Microscopic observations of spores were carried out as follows. *B. subtilis* spores were observed on agarose pads (1% Agarose in H_2_O) using a Zeiss Axioskop 2 microscope (Zeiss, Germany) configurated for phase-contrast observation with a 100x immersion objective (Plan-NEOFLUAR, 100x/1,30 oil, Ph3). Spores were negatively stained with India ink as described previously (Abe et al., 2014) and they were observed in the configuration described above. Fluorescence and localization of CotY-GFP and CgeA-GFP fusion proteins on the spore surface were observed using the Zeiss Axioskop 2 microscope with the appropriate filter set (Filter set 09, BP 450–490 FT510 LP515) and a 100x immersion objective (αPlan-APOCHROMAT, 100x/1,46 Oil).

### 2.5. Observation of sporulating cells by confocal microscopy

Sporulating cells were collected 8 and 24 h (t8 and t24) after the transition to the stationary phase. They were fixed in a 4% formaldehyde solution in phosphate buffer saline (PBS) for 7 min and washed in PBS before being suspended in GTE buffer (50 mM glucose, 10 mM EDTA at pH 8, and 20 mM tris–HCl at pH 8). After fixation, the cells were kept at 4°C until observations. The membranes of fixated cells were stained by adding FM 4–64 (SynaptoRed C2, Sigma-Aldrich, S6689) at a working concentration of 10 μM for 30 min at room temperature in the dark. Then, the cells were washed, concentrated by centrifugation, and transferred on 1% agarose pads. Microscopic observations were performed by using the inverted confocal laser scanning microscope LSM780 (Zeiss, Germany) with an excitation wavelength of 514 nm, and by detecting the signal at 650–740 nm using a 63x immersion objective (αPlan-APOCHROMAT, 63x/1,4 Oil).

### 2.6. Experiments on mature spores

To verify that the conditions tested affected the sporulating bacteria and not the mature spores, the same conditions were tested on mature spores. For this purpose, spores were first produced in optimal condition (Spo8) and then subjected to the -CaCl_2_, -MgSO_4_, -O_2_, -O_2_ + CO_2_, peracetic acid, H_2_O_2_ or pH 5 conditions, except that Spo8 was modified by removing the NB to prevent spore germination. Then, mature spores were incubated at 30°C under agitation (220 rpm). After 3 days, spores were harvested by centrifugation (2,500 *g* for 15 min, 4°C) and washed five times in chilled sterile water (2,500 *g* for 15 min, 4°C) before being stored at 4°C in sterile water until use. Spores were then tested for their surface hydrophilic/hydrophobic properties.

### 2.7. Crust extraction and analysis

The crust was removed from the spore surface by three successive passages of spore suspensions through a French press (SLM instruments, Urbana, IL) at 20000 psi. Spores were separated from the crust by centrifugation (6,000 *g*, 15 min, 4°C) and the supernatant (the crust extract) was kept at −20°C until use.

Release and derivation of the crust monosaccharides were adapted from previous studies (Anumula, 1994; Windwarder et al., 2016). Crust extracts were dried by lyophilization and hydrolyzed at 100°C for 4 h in 4 M trifluoroacetic acid (TFA) to release the monosaccharides. Samples were dried under a nitrogen flow at 40°C in an evaporator (puriVap-6, Interchim) and the dried pellets were suspended in a 50:50 (v/v) mix of a fresh 1% sodium acetate solution in methanol and a fresh solution containing 30 mg of anthranilic acid (AA) and 20 mg of sodium cyanoborohydride in a solution of 2% boric acid and 4% sodium acetate in methanol. The monosaccharides were then coupled to the anthranilic acid by heating the samples at 80°C for 1 h. Release and derivation of Leg were performed as described previously (Dubois et al., 2020). Crust extracts were dried by lyophilization and hydrolyzed at 80°C for 2 h in 0.1 M TFA to release the nonulosonic acids. Nonulosonic acids were subsequently coupled to 1,2-diamino-4,5-methylenedioxybenzene (DMB) by heating the samples at 50°C for 2 h in the dark in 7 mM DMB, 1 M β-mercaptoethanol, 18 mM sodium hydrosulfite in 0.02 mM trifluoroacetic acid.

The monosaccharide derivatives were separated on a C18 reverse-phase HPLC column (Thermo Scientific, ODS Hypersil, 5 μm, 4.6 × 250 mm) at a flow rate of 1 mL/min following a protocol adapted from Windwarder et al. (2016). The solvent A was 80 mM formic acid adjusted to pH 3.4 with ammonia and the solvent B was a mixture of acetonitrile:solvent A (80:20, v/v). The elution program was a gradient from 9.5 to 16% of solvent B for 20 min followed by a second gradient from 16 to 36% for 20 min. Fluorescence was measured with a Waters 2,475 fluorimeter (λ_exc_ = 230 nm, λ_em_ = 425 nm). A standard containing GlcN, galactose (Gal), Glc, Rha, and Qui was used to estimate the amount of the different monosaccharides. The nonulosonic acid derivatives were separated isocratically at a flow rate of 0.8 mL/min on a C18 reverse phase HPLC column (Waters, 3.5 μm, 4.6 × 150 mm) using a solvent mixture of acetonitrile:methanol:water (7:9:84, v/v/v) and they were detected by a fluorimeter (Waters 2,475, λ_exc_ = 373 nm, λ_em_ = 448 nm). The Leg amount was estimated by measuring the area of peak 1 and by reporting this area to a standard range of Neu5Ac.

Gas chromatography–mass spectrometry (GC–MS) experiments were performed as previously described (Faille et al., 2014).

The relative amount of CotY or CgeA in the crust was evaluated by using *B. subtilis* 168 *amyE*::*cotY*-GFP strain or *B. subtilis* 168 *amyE*::*cgeA*-GFP strain in the sporulation conditions described above. After washing in chilled water, spores were diluted in 1X PBS buffer and gently vortexed. Then, the fluorescence of spores was evaluated by flow-cytometry with the BD Acuri C6 Plus flow-cytometer (BD biosciences, USA) using a 488 nm laser and 585/40 bandpass filter to detect FITC fluorescence. The following voltage values were used: 200 V for the forward scatter, 250 V for the side scatter, and 400 V for the FITC channel according to the manufacturer’s recommendations. The cytometer was programmed to count 40,000 events and the spore suspension was diluted to obtain less than 2,500 events per second.

### 2.8. Transcriptional analysis of genes involved in crust biosynthesis

The sporulating cells were collected and harvested by centrifugation (5,000 *g*, 15 min at 4°C) 8 h after the transition to the stationary phase. RNA extraction, cDNA synthesis, amplification, and detection were performed as previously described (Dubois et al., 2016) except that the cell lysis was performed using screw tubes containing 150 mg of glass beads (acid-washed, Sigma-Aldrich, G1277-500G,) and a MiniBeadBeater-16 (Biospec Products). The tubes were shaken for 2 min and cooled on ice for 2 min. The previous step was repeated once and the lysates were centrifuged at 12,000 *g* for 10 min at 4°C before transferring to a new 2 mL tube. The quantity of each cDNA was normalized to the quantity of the cDNA of the DNA polymerase III gene (*polC*, BSU16580) (Supplementary Table 2). The relative change in gene expression was recorded as the ratio to normalized target concentrations (threshold cycle [∆∆*CT*]) (Livak and Schmittgen, 2001).

## 3. Results

To investigate the influence of the environmental conditions encountered by *B. subtilis* during sporulation on spore surface and adhesion properties, sporulation conditions were modified before bacteria inoculation or 2 h after the transition to the stationary phase (Table 1; Supplementary Figure 1). These 13 conditions were tested to evaluate the influence of depletion in divalent cations (-CaCl_2_ or -MgSO_4_), oxidative stresses (peracetic acid or H_2_O_2_), atmospheres (-O_2_ or -O_2_ + CO_2_), pH (pH 9 or pH 5), preservatives (benzoic acid or nitrites), UV as well as cleaning and disinfecting agents (RBS or Benzalkonium Chloride [BAC]). The chemicals added were used at the maximum concentration that did not reduce the total number of CFU at the end of sporulation to avoid major structural changes in the internal layers of spores.

### 3.1. Influence of sporulation conditions on refractivity, heat resistance, and germination rate of *Bacillus subtilis* 168 spores

It was first investigated whether the sporulation conditions affected the total number of CFU as well as some key characteristics of spores, i.e., their refractivity, heat resistance, and ability to germinate. The total number of CFU at the end of sporulation was increased in the -O_2_, -O_2_ + CO_2_, peracetic acid, and pH 5 conditions while it was reduced by a logarithmic factor of 0.45 in the -CaCl_2_ condition compared to the Spo8 condition (Supplementary Table 3). None of the sporulation conditions changed the spore characteristics except for those obtained in the -CaCl_2_ condition (Supplementary Table 3; Supplementary Figure 2). In this latter condition, the percentage of heat resistant spores was unchanged but 35% of spores were non-refractive and the ability of spores to germinate was reduced by 40% compared to the Spo8 condition. These results suggest that in the -CaCl_2_ condition, a part of the cells that engage in the sporulation process do not release viable spores. However, the viable spores produced in this condition keep their heat resistance and most of them can germinate. Therefore, this condition was kept for subsequent experiments.

### 3.2. Divalent cations depletion, oxidative stresses, lower oxygen availability and acidic pH during sporulation promote spore adhesion

**Figure 1:**
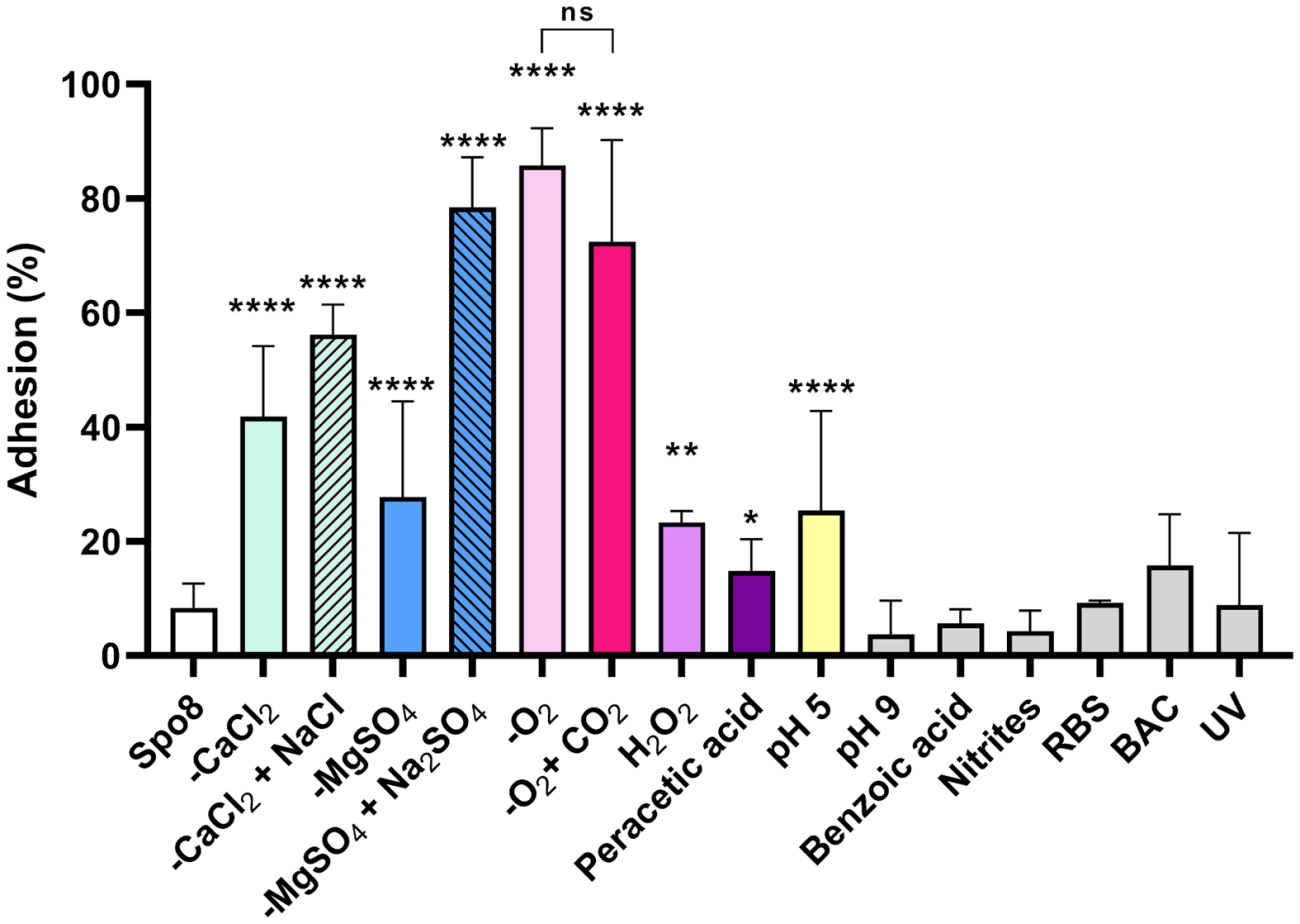
Influence of sporulation conditions on spore adhesion to polypropylene. Adhesion (%) was determined after 10 successive adhesion steps of the spores to polypropylene tubes. The whole adhesion kinetics are presented in Supplementary Figure 3. Error bars represent the SDs of the means. ns, not significant. *p ≤ 0.05; **p ≤ 0.01; ***p ≤ 0.001; ****p ≤ 0.0001 for each condition versus Spo8 by Mann–Whitney.

To evaluate the consequences of the sporulation conditions on spore adhesion, an adhesion test on polypropylene was performed. *B. subtilis* 168 spores produced in 7 of the 13 conditions tested were significantly more adherent to polypropylene than those produced in Spo8 (Figure 1; Supplementary Figure 3). This is particularly true for spores produced in -CaCl_2_ or -MgSO_4_ conditions. Replacement of CaCl_2_ or MgSO_4_ by NaCl or Na_2_SO_4_ in the sporulation medium also led to the production of more adherent spores indicating that the low concentrations of Mg^2+^ or Ca^2+^ rather than the associated anions (SO_4_^2−^ or Cl^−^) were responsible for the increased adhesion of spores. Bacteria that sporulated in a modified atmosphere (-O_2_ and -O_2_ + CO_2_), at acidic pH (pH 5) and to a lesser extent those that were exposed to oxidative stress (H_2_O_2_, peracetic acid) also produced spores that were more adherent than spores produced in Spo8. Interestingly, there was no significant difference in adhesion between spores produced in -O_2_ and -O_2_ + CO_2_ conditions, which would indicate that the decrease in O_2_ and not the increase in CO_2_ concentration during sporulation is responsible for this increase in adhesion. To verify that the increase in adhesion was not due to a delay in the sporulation process, sporulating cells were observed by confocal microscopy 8 and 24 h after the transition to the stationary phase. No delay in the sporulation process was observed (Supplementary Figure 4).

### 3.3. Increased spore adhesion is correlated with an increase of spore hydrophobicity

Since the spores are metabolically dormant, the increase in spore adhesion can only be explained by modifications of the spore surface properties including hydrophobicity and charge. Thus, the surface hydrophilic/hydrophobic property and the surface charge of spores were evaluated by MATH and zetametry (Table 2; Supplementary Figure 5). All the spores obtained under the sporulation conditions that led to an increase in spore adhesion were more hydrophobic, whereas no modification of this character could be demonstrated for spores whose adhesion was not affected. The correlation factor between spore hydrophobicity and spore adhesion to polypropylene was 0.71 indicating that the increased adhesion of spores is at least in part due to an increase of spore’s hydrophobicity (Supplementary Figures 6A,C). By contrast, whatever the sporulation condition, the global charge of spores at a neutral pH was similar (around −50 mV), except for spores produced at pH 5 and in the presence of benzoic acid (Table 2). However, the spore electronegativity would not play a significant role in their ability to adhere to polypropylene (correlation factor = −0.14) (Supplementary Figures 6B,C). It is now well documented that alteration of the crust generally leads to an increased hydrophobicity and adhesion of spores (Faille et al., 2014; Shuster et al., 2019a,b; Dubois et al., 2020). Therefore, our results suggest that environmental conditions encountered during sporulation affect crust composition and/or structure.

**Table 2:** Influence of the sporulation conditions on surface properties of spores.

Sporulation condition	Hydrophobicity (LogK)	Zeta potential (mV)
Spo8	0.60 ± 0.96	-52.48 ± 5.22
-CaCl_2_	2.24 ± 0.80***	-49.48 ± 1.66
-MgSO_4_	2.32 ± 0.73***	-51.68 ± 0.83
-O_2_	3.60 ± 1.03****	-50.79 ± 1.06
-O_2_ + CO_2_	4.30 ± 0.52****	-50.03 ± 2.36
H_2_O_2_	1.49 ± 0.83**	-55.98 ± 5.20
Peracetic acid	2.47 ± 1.30***	-50.98 ± 4.75
pH 5	2.54 ± 0.81****	-56.52 ± 3.17*
pH 9	1.00 ± 0.44	-49.89 ± 2.97
Benzoic Acid	1.22 ± 0.70	-50.30 ± 1.01*
Nitrites	0.29 ± 0.19	-50.59 ± 2.07
RBS	0.21 ± 0.44	-49.19 ± 3.69
BAC	0.85 ± 0.66	-55.06 ± 5.26
UV	1.23 ± 0.48	-56.37 ± 8.83

The surface hydrophilic/hydrophobic property and the surface charge of spores at pH 7.0 were evaluated by MATH and zetametry assays. The data presented in the table are the means with SDs. *p ≤ 0.05; **p ≤ 0.01; ***p ≤ 0.001; ****p ≤ 0.0001 for each condition versus Spo8 by Mann–Whitney or *t*-test.

### 3.4. The modification of the spore surface properties occurs during sporulation

We investigated whether the same environmental conditions applied to mature spores affected their surface properties, i.e., whether the changes in spore surface observed above were due to phenomena occurring during sporulation or after the release of the mature spores. For this purpose, mature spores produced in Spo8 condition were subjected to the seven conditions shown to modify the adhesion and hydrophobicity of spores when applied during sporulation. Then, the hydrophobicity of spores was evaluated by MATH (Table 3). The seven conditions applied to the mature spores did not alter their hydrophobic character, which confirmed that the changes observed above were indeed the result of phenomena occurring during sporulation.

**Table 3:** Influence of environmental conditions on surface hydrophilic/hydrophobic property of mature spores.

Sporulation condition	Hydrophobicity (LogK)
Spo8 -NB	1.25 ± 1.04
-CaCl_2_	0.74 ± 1.14
-MgSO_4_	0.76 ± 1.21
-O_2_	0.68 ± 0.43
-O_2_ + CO_2_	0.54 ± 0.59
Peracetic acid	1.70 ± 0.62
H_2_O_2_	1.53 ± 0.95
pH 5	1.52 ± 0.96

The conditions affecting the hydrophobicity and adhesion of spores when applied during sporulation were tested on mature spores. Spores were produced in optimal condition (Spo8), washed and suspended in a Spo8 medium modified by removing the NB to prevent spore germination (Spo8 -NB) and subjected to the -CaCl_2_, -MgSO_4_, -O_2_, -O_2_ + CO_2_, peracetic acid, H_2_O_2_ or pH 5 conditions. After 72 h of incubation, the surface hydrophilic/hydrophobic property of spores was evaluated by MATH assays. The data presented in the table are the means with SDs of at least three independent experiments. None of the conditions tested were significantly different from the Spo8 -NB condition.

### 3.5. The crust glycans of spores produced in divalent cations depletion or oxygen-limited conditions are impaired

**Figure 2:**
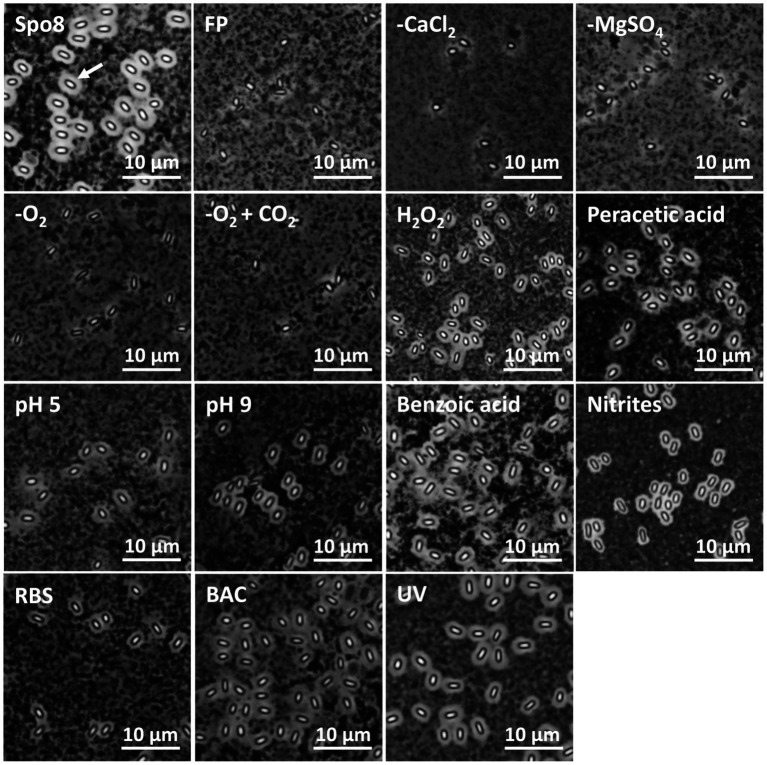
Observation of spores by phase contrast microscopy after India ink staining. FP: Spores after a French press treatment (negative control). A white halo around the spores (white arrow) indicates the presence of crust glycans.

To evaluate the impact of the sporulation conditions on crust structure, spores were observed in phase-contrast microscopy after India ink staining (Figure 2). Indeed, the India ink staining enables the observation of the crust glycans around *B. subtilis* spores. Spores produced in Spo8 (positive control) showed a white halo surrounding the outer coat of spores (white arrow), which reflects the presence of the crust glycans. Conversely, no white halo was observed when spores produced in Spo8 were passed through a French press, this treatment resulting in the removal of the crust without damaging the coat (negative control) (Faille et al., 2014). A white halo was observed around spores produced in the H_2_O_2_, peracetic acid, pH 5, pH 9, benzoic acid, nitrites, BAC, RBS, and UV conditions. In contrast, no halo was observed around spores produced in the -CaCl_2_, -MgSO_4_, -O_2_, and -O_2_ + CO_2_ conditions indicating that the crust glycans are deeply impaired in these conditions.

### 3.6. Divalent cation depletion or lower oxygen availability during sporulation change the crust composition in monosaccharides

**Figure 3:**
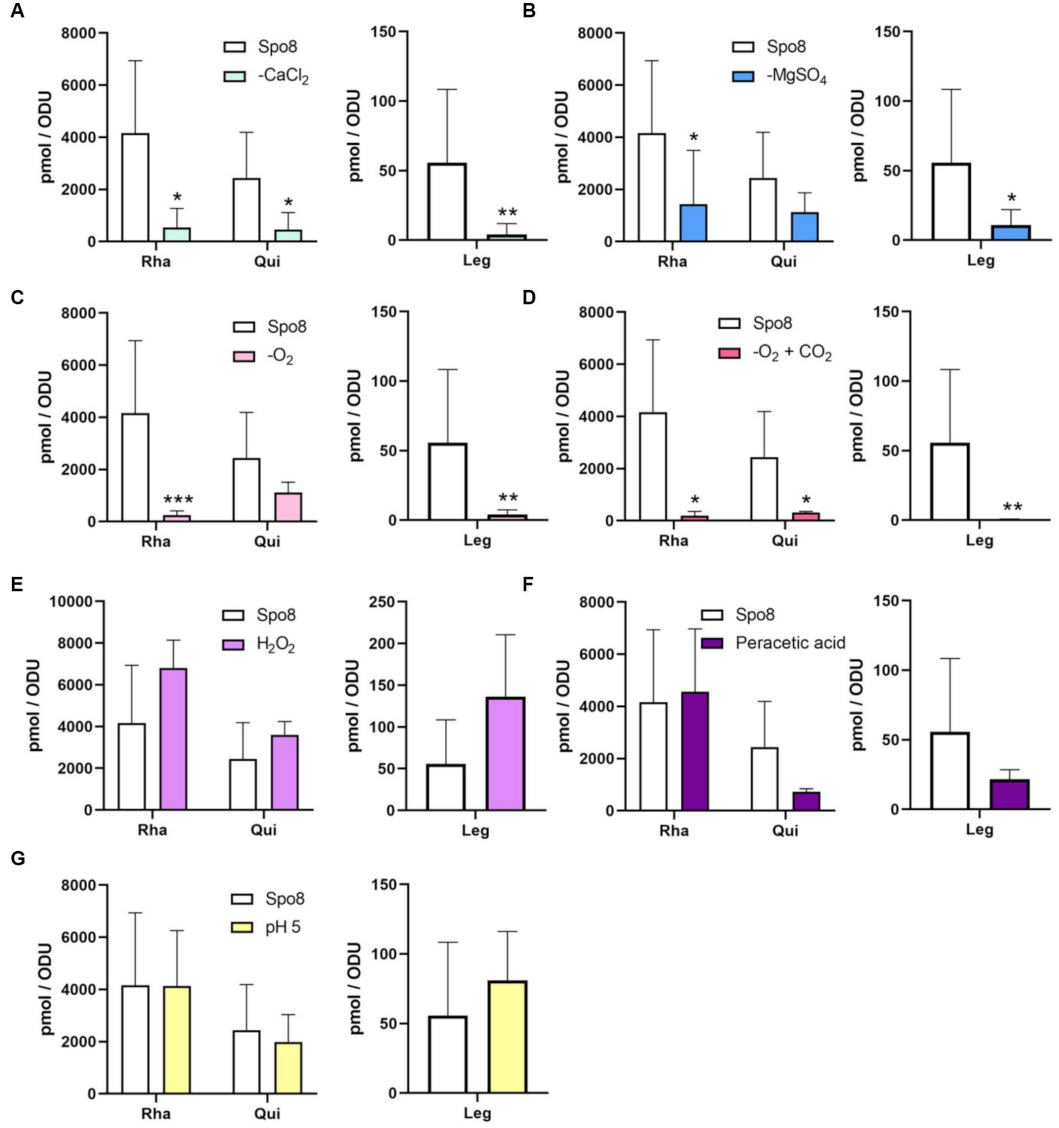
Influence of sporulation conditions on the monosaccharide composition of the crust. The relative amounts of Rha, Qui, and Leg in the crust were evaluated by RP-HPLC-FL. The experiments were performed on the crust of spores released from *B. subtilis* 168 cells produced in Spo8 and in the sporulation conditions shown to affect spore surface properties: -CaCl_2_ **(A)**, -MgSO_4_ **(B)**, -O_2_ **(C)**, -O_2_ + CO_2_ **(D)**, H_2_O_2_ **(E)**, peracetic acid **(F)**, and pH 5 **(G)**. The results were standardized by the OD_600nm_ of the spore preparations. Error bars represent the SDs of the means. *p ≤ 0.05; **p ≤ 0.01; ***p ≤ 0.001 for each condition versus Spo8 by Mann–Whitney.

The monosaccharide composition of the crust was further investigated. Spores were mechanically treated with a French press to remove the crust. Then, the crust glycans were hydrolyzed and the released monosaccharides were derivatized by AA, a fluorogenic reagent that shows high specificity for monosaccharides. The AA-complexed monosaccharides were then analyzed by reverse-phase high-performance liquid chromatography coupled to a fluorescence detector (RP-HPLC-FL) (Anumula, 1994; Windwarder et al., 2016). In the Spo8 condition, the chromatogram of RP-HPLC-FL showed 12 major peaks (Supplementary Figure 7A). By performing co-injections with standards four peaks were associated with monosaccharides previously identified in the crust: GlcN with a retention time of about 11.8 min, Glc with a retention time of about 16.7 min, Rha with a retention time of about 20.8 min and Qui with a retention time of about 25.4 min (Supplementary Figure 7B; Faille et al., 2014). The peak with a retention of 15.9 min co-eluted with the Gal standard and GC–MS experiments confirmed the presence of Gal in the crust of *B. subtilis* spores (Supplementary Figures 7A–F). Using RP-HPLC-FL, the influence of the conditions shown to increase spore adhesion on the amount of the five monosaccharides identified in the crust was evaluated. In all the conditions tested, there was no significant change in the amounts of GlcN, Gal, and Glc compared to the Spo8 condition (Supplementary Figures 8A–G). The composition of Rha and Qui was also not modified in the crust of spores produced in the H_2_O_2_, peracetic acid, and pH 5 conditions (Figures 3E–G). Conversely, the amount of Rha was lowered in the crust of spores produced in the -CaCl_2_, -MgSO_4_, -O_2_ and -O_2_ + CO_2_ conditions, and the amount of Qui was lowered in the crust of spores produced in the -CaCl_2_ and -O_2_ + CO_2_ conditions (Figures 3A–D).

### 3.7. Sporulating cells exposed to divalent cations depletion or lower oxygen availability produce spores with a lower amount of leg in the crust

To evaluate the impact of the sporulation conditions on the amount of Leg on the spore surface, crust samples were hydrolyzed, and nonulosonic acids were derivatized by DMB before being analyzed by RP-HPLC-FL. The chromatogram obtained in the Spo8 condition showed three major peaks with retention times of ~8.3 min (peak 1), 14.5 min (peak 2), and 20.4 min (peak 3). The area of peak 1, which was previously identified as Leg, was used to estimate the Leg amount in the crust of *B. subtilis* spores (Supplementary Figure 9; Dubois et al., 2020). The amount of Leg was significantly reduced in the -CaCl_2_, -MgSO_4_, -O_2_, and -O_2_ + CO_2_ conditions compared to the Spo8 condition (Figures 3A–D). In contrast, it was not significantly different in the H_2_O_2_, peracetic acid, and pH 5 conditions (Figures 3E–G).

### 3.8. The protein backbone of the crust of spores produced in the oxygen-limited and H_2_O_2_ conditions is impaired

**Figure 4:**
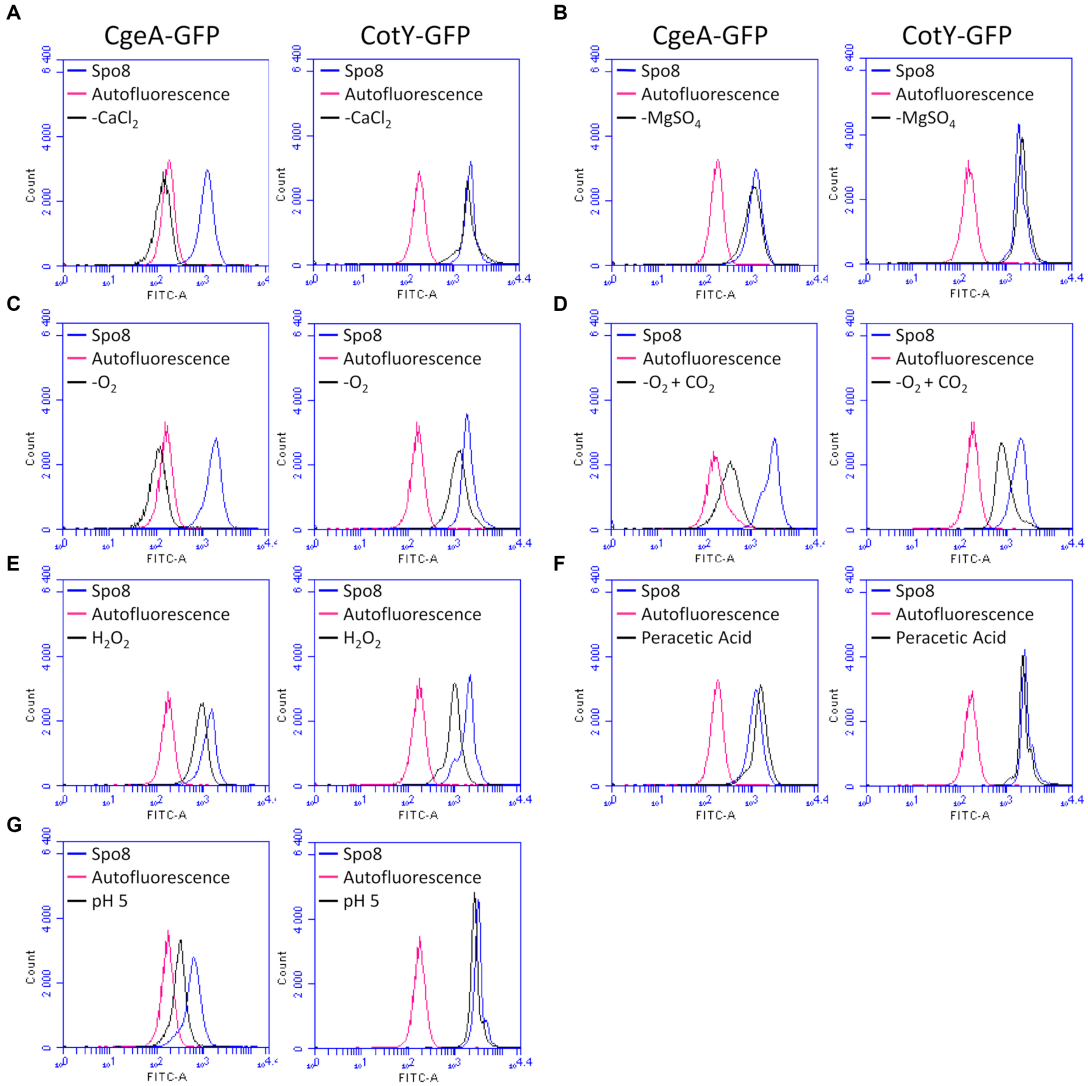
Influence of sporulation conditions on the relative amount of CgeA and CotY in the crust. Fluorescence of spores of the *B. subtilis 168 amyE*::*cgeA*-GFP and *B. subtilis* 168 *amyE::cotY*-GFP strains was measured by flow cytometry after sporulation of both strains in Spo8 and in the sporulation conditions shown to affect spore surface properties: -CaCl_2_ **(A)**, -MgSO_4_ **(B)**, -O_2_ **(C)**, -O_2_ + CO_2_ **(D)**, H_2_O_2_ **(E)**, peracetic acid **(F)** and pH 5 **(G)**. The fluorescence of spores is proportional to the amount of CgeA-GFP and CotY-GFP fusion proteins on the spore surface. Autofluorescence of the *B. subtilis* 168 strain is represented in red in all the panels (negative control). The data are presented as overlaid histograms that represent the distribution of fluorescence per cell of the same number of events.

To assess the effect of the sporulation conditions on crust proteins, the *cotY* and *cgeA* genes with their respective promoter were fused to the gene encoding the green fluorescent protein (GFP). Fusions were then integrated at the *amyE* locus of the *B. subtilis* 168 strain to obtain the *B. subtilis* 168 *amyE*::*cotY*-GFP and *B. subtilis* 168 *amyE*::*cgeA*-GFP. These two strains produced CotY-GFP and CgeA-GFP fusion proteins, respectively. The CotY-GFP fusion protein was used here as a marker of the integrity of the protein backbone of the crust while the CgeA-GFP fusion protein was used to detect any change in the composition and/or structure of the crust proteins. Spores of the *B. subtilis* 168, *B. subtilis* 168 *amyE*::*cotY*-GFP, and *B. subtilis* 168 *amyE*::*cgeA*-GFP strains were produced in the Spo8 condition and the fluorescence of spores was evaluated by fluorescence microscopy. Spores of the *B. subtilis* 168 strain were slightly auto-fluorescent at the excitation wavelength of GFP and a strong fluorescence signal was observed on the spore surface of the *B. subtilis* 168 *amyE*::*cotY*-GFP and *B. subtilis* 168 *amyE*::*cgeA*-GFP strains indicating that CotY-GFP and CgeA-GFP fusion proteins were properly expressed and localized (Supplementary Figure 10). Then, spores of the *B. subtilis* 168 *amyE*::*cotY*-GFP and *B. subtilis* 168 *amyE*::*cgeA*-GFP strains were produced in the seven sporulation conditions affecting surface properties and fluorescence of spores was measured by flow-cytometry (Figure 4; Supplementary Figures 11A,B). In the -O_2_, -O_2_ + CO_2_, and H_2_O_2_ sporulation conditions, the fluorescence of spores of *B. subtilis* 168 *amyE*::*cotY*-GFP strain was significantly lower than that of spores of the same strain obtained in the Spo8 condition. These results indicate that these sporulation conditions lead to a decrease in the relative amount of CotY in the crust. The decrease in spore fluorescence in the -O_2_ and -O_2_ + CO_2_ conditions was not due to a decrease in GFP fluorescence caused by a lower oxygen availability during sporulation (Supplementary Figure 11C). In the -CaCl_2_, -O_2_, -O_2_ + CO_2_, H_2_O_2_, and pH 5 sporulation conditions, fluorescence of spores of the *B. subtilis* 168 *amyE*::*cgeA*-GFP strain was lower than that of spores of the same strain obtained in the Spo8 condition indicating a decrease in the relative amount of CgeA in the crust of spores produced in these conditions.

### 3.9. Transcription of genes involved in crust biosynthesis is decreased during late sporulation in oxygen-limited and H_2_O_2_ conditions

**Figure 5:**
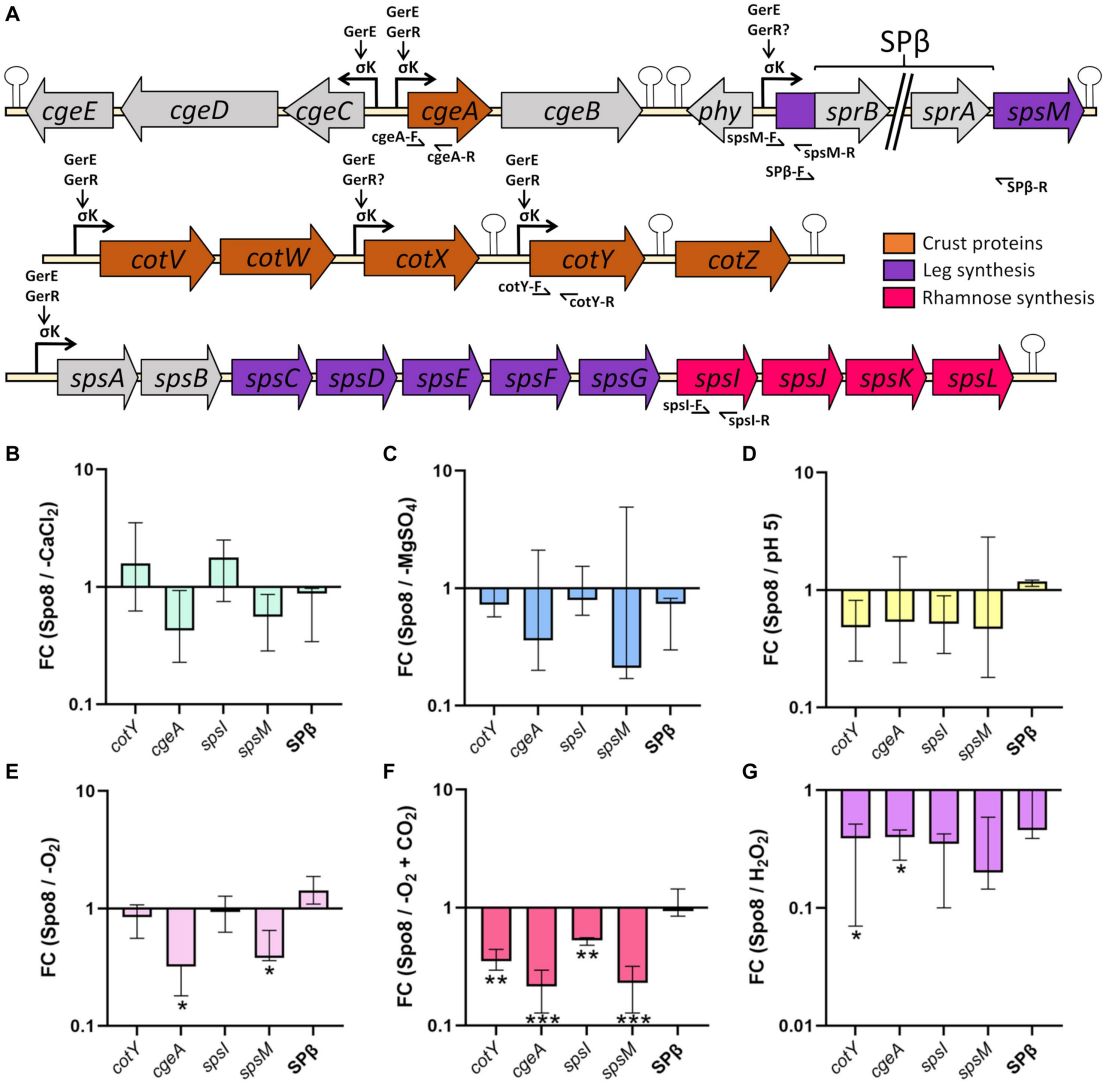
Influence of sporulation conditions on the transcription of genes involved in crust synthesis. **(A)** Schematic representation of the genetic organization of the *cge*, *cot*, and *sps* genes involved in crust synthesis. Broken arrows, arrows and, stem-loop, respectively, represent the σ^K^ dependent promoters, direct or indirect activation of transcription by a transcriptional regulator and transcriptional terminators defined previously (Zhang et al., 1994; Roels and Losick, 1995; Eichenberger et al., 2004; Kuwana et al., 2005; Nicolas et al., 2012; Cangiano et al., 2014; Arrieta-Ortiz et al., 2015). Primers used for qRT-PCR are indicated by half arrows. Relative transcript levels of the *cotY*, *cgeA*, *spsI*, and *spsM* genes and SPβ prophage excision were evaluated by qRT-PCR in the late stationary phase (t8). SPβ prophage excision was assessed using primers flanking the prophage (SPβ-F/SPβ-R). Relative transcript levels were calculated as the ratio of the mRNA level (arbitrary units) of each gene in the sporulation conditions shown to affect spore surface properties: -CaCl_2_ **(B)**, -MgSO_4_ **(C)**, pH 5 **(D)**, -O_2_ **(E)**, -O_2_ + CO_2_ **(F)** and H_2_O_2_ **(G)**, compared to that obtained in the Spo8 condition. The fold change (FC) was calculated by the ΔΔCT method using the *polC* gene for normalization. Error bars represent the median with the interquartile range. *p ≤ 0.05; **p ≤ 0.01; ***p ≤ 0.001 for each condition versus Spo8 by *t*-test.

We have shown above that -CaCl_2_, -MgSO_4_, -O_2_, -O_2_ + CO_2_, H_2_O_2_, and pH 5 sporulation conditions modify the glycan and/or protein composition of the crust of the released spores. To determine whether these crust modifications are due to transcriptional regulation of genes involved in crust synthesis, qRT-PCR experiments were performed on RNA extracted from sporulating cells in the late stationary phase (8 h after the transition to the stationary phase). The genes encoding crust proteins (*cotY* and *cgeA*) and the genes encoding the first enzyme of the Rha and Leg biosynthesis pathway (*spsI* and *spsM*, respectively) were targeted (Figure 5A). In addition, a primer pair flanking the SPβ prophage was tested to evaluate the influence of sporulation conditions on SPβ prophage excision. In the -CaCl_2_, -MgSO_4,_ and pH 5 conditions, transcription of the four genes was unchanged compared to the Spo8 condition (Figures 5B–D). In contrast, transcription of the *cgeA* and *spsM* genes was decreased in the -O_2_ condition, and transcription of all the genes tested was reduced in the -O_2_ + CO_2_ condition (Figures 5E,F). Finally, in the H_2_O_2_ condition, transcription of *cotY* and *cgeA* genes was decreased compared to the Spo8 condition (Figure 5G). Our results also showed that prophage excision is not changed whatever the condition tested. These results suggest that the changes in spore surface properties obtained in the -O_2_, -O_2_ + CO_2,_ and H_2_O_2_ conditions are, at least in part due to a decrease in the transcription of genes involved in crust synthesis during late sporulation.

## 4. Discussion

In optimized growth conditions, e.g., in laboratory conditions, sporulation of *B. subtilis* is triggered when the cells enter in the transition to the stationary phase, and it takes 8–10 h to complete with the release of a mature spore. Therefore, sporulation is a long process and its duration can be further extended in suboptimal growth conditions such as those found in natural and industrial environments. In these environments, spore-forming bacteria often encounter unfavorable conditions for growth and sporulation, such as low or high temperatures and pH, which can modulate sporulation rate and length, but also the properties of mature spores (Bressuire-Isoard et al., 2018). Many works have been reported in the literature on the influence of sporulation conditions on spore resistance, notably to extreme temperatures and UV radiations. Other works have concerned the structure of the spores, e.g., the water or dipicolinic acid content, the cortex peptidoglycan structure, the fatty acid composition, or the coat protein profile, which are probably at least partly related to changes in spore resistance (Abhyankar et al., 2016; Setlow and Christie, 2023). Conversely, only a few works have been reported on the surface properties of spores produced in different environmental conditions, despite their major role in surface contamination and therefore their possible role in the risk of further dissemination, e.g., in agri-food industries or medical environments.

This study evaluated the impact of 13 sporulation conditions likely to be encountered in real environments, on *B. subtilis* spore surface and adhesion properties. Some of these conditions, such as reduced cation availability, or acidic pH, have previously been shown to affect spore resistance (Cazemier et al., 2001; Baweja et al., 2008). In contrast, the conditions used to limit the growth and/or survival of bacteria, such as preservatives, detergents, and disinfectants seem not to have been investigated in the literature. Out of the 13 sporulation conditions, 7 led to the release of spores that are more hydrophobic and adherent, while the other conditions did not affect these spore properties. Concerning the lack of effect of the detergent and antibacterial treatments, it can be assumed that this would be due to the selected relatively mild conditions, so as not to impede sporulation. Six of these seven sporulation conditions leading to more adherent spores can be categorized into three groups according to the nature of the stresses (Table 4): depletion in divalent cation (-CaCl_2_ and -MgSO_4_), lower oxygen availability (-O_2_ and -O_2_ + CO_2_) and oxidative stress (H_2_O_2_ and peracetic acid). These results suggest that in their ecological niches and industrial environments, where growth and sporulation conditions are not optimal, the bacteria of the *B. subtilis* group probably produce spores that are often more hydrophobic and adherent than spores obtained in optimized laboratory conditions.

**Table 4:** Summary table of the results.

		Adhesion	Hydrophobicity	Halo	Rha	Qui	Leg	CotY	CgeA	Transcription *
Sporulation condition	Group									
-CaCl_2_	Depletion in cation	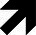	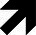	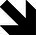	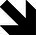	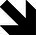	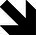	–	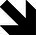	–
-MgSO_4_		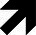	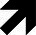	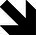	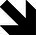	–	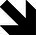	–	–	–
-O_2_	Lower oxygenation	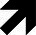	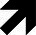	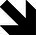	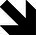	–	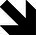	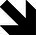	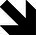	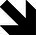
-O_2_ + CO_2_		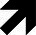	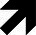	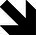	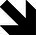	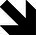	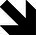	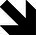	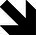	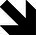
H_2_O_2_	Oxidative stress	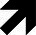	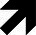	–	–	–	–	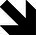	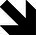	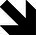
Peracetic acid		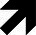	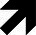	–	–	–	–	–	–	–
pH 5		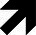	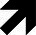	–	–	–	–	–	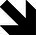	–

The column adhesion refers to the results of polypropylene adhesion tests, the column hydrophobicity refers to the results of MATH, the column Halo refers to microscopic observations of spores after India ink staining, the columns Rha, Qui, and Leg refer to RP-HPLC-FL experiments, the columns CgeA and CotY refer to the cytometry experiments and the column transcription refers to the qRT-PCR experiments. An upward arrow indicates an increase and a downward arrow indicates a decrease relative to the Spo8 condition. *: A downward arrow indicates that transcription of at least one of the 4 targeted genes decreased.

The surface and adhesion properties of spores depend on their outermost layer which is in contact with surfaces in the environment. In most strains of the *B. subtilis* group, the crust is the outermost layer of spores and it has been shown that its mechanical removal makes *B. subtilis* spores hydrophobic and more adherent to stainless steel (Faille et al., 2014; Dubois et al., 2020). Therefore, we investigated the impact of the sporulation conditions on the crust structure and composition. Spores produced in the different conditions shown to affect their surface properties were observed under microscope after staining with India ink, a stain that does not penetrate the mucous layer formed by the crust glycans, producing a white halo around the spores (Abe et al., 2014; Shuster et al., 2019b). In the divalent cation depletion (-CaCl_2_ or -MgSO_4_) and oxygen-limited (-O_2_ or -O_2_ + CO_2_) conditions, the crust glycans were impaired confirming the negative impact of these sporulation conditions on crust glycans synthesis and/or assembly on the spore surface. In contrast, the crust glycans of spores obtained from sporulating cells exposed to oxidative stresses (H_2_O_2_ and peracetic acid) or low pH (pH 5) seemed unaffected.

To determine if these observations were related to a decrease in Leg or one or more monosaccharides, the composition of the crust of these spores was implemented. We first confirmed that the crust contains Rha, Glc, Qui, GlcN, and Leg (Faille et al., 2014; Dubois et al., 2020) and we also demonstrated the presence of Gal (Supplementary Figure 7). A reduced amount of Rha, Qui, and Leg was observed on some spores, the alteration of the crust of which was revealed by staining with India ink, while no changes could be evidenced on the spores still surrounded with a clear halo. Interestingly, the relative amounts of Rha and Leg decreased concomitantly in divalent cations depletion as well as oxygen-limited conditions. This result is consistent with a previous study that showed that inactivation of the Leg biosynthesis pathway abolishes Leg production but also decreases the amount of Rha in the crust (Dubois et al., 2020). These data suggest that Rha and Leg are part of a same glycan. It was shown previously that the Leg biosynthesis pathway is required for crust assembly and spores from mutants of this pathway are more hydrophobic and adherent to stainless steel (Dubois et al., 2020). Similarly, it was shown by transmission electron microscopy that the crust of a rhamnose-deficient mutant (∆*spsI*) is present around *B. subtilis* spores but it is barely detectable and spores of this mutant were more hydrophobic than spores of a rhamnose producing strain (Shuster et al., 2019b). These data strongly suggest that Rha and Leg play an important role in spore surface and adhesion properties. Therefore, it seems likely that the increased adhesion of spores obtained after sporulation in divalent cations depletion or oxygen-limited conditions is due to a decrease in the amount of a glycan containing Rha and Leg in the crust. On the other hand, the increased adhesion of spores produced under oxidizing conditions cannot be explained by the modification of the crust glycans.

We also evaluated the impact of the sporulation conditions on the relative amount of the CotY and CgeA proteins in the crust. CotY is one of the morphogenetic proteins and the main structural protein of the crust of *B. subtilis* spores (Bartels et al., 2019). Indeed, expressed in *E. coli*, CotY can arrange intracellularly into highly stable honeycomb-like structures through processes of self-assembly (Jiang et al., 2015). Therefore, the CotY-GFP fusion protein was used as a marker of the integrity of the protein backbone of the crust. In contrast, CgeA is not required for the assembly or the correct localization of the other crust proteins and it relies on all other crust proteins for proper localization (Bartels et al., 2019; Shuster et al., 2019a). It was deduced that CgeA is at the bottom of the crust assembly hierarchy (Bartels et al., 2019; Shuster et al., 2019a). Therefore, the CgeA-GFP fusion protein was used to detect any change in the composition and/or structure of the crust proteins. Our results showed that all the sporulation conditions tested changed the protein composition of the crust except for -MgSO_4_ and peracetic acid conditions. In the -CaCl_2_ and pH 5 conditions, the changes in the crust appear to be moderate, as only the relative amount of CgeA decreased. In contrast, when sporulation was perturbed by hydrogen peroxide stress or oxygen-limited conditions, the relative amount of both CgeA and CotY decreased suggesting that the protein backbone is impaired. The underlying molecular mechanisms that explain the changes in crust composition and structure will have to be studied but they probably interfere with crust synthesis and/or assembly. Nevertheless, it is also possible that defects in crust assembly were the indirect consequence of defects in the inner layers of spores, especially the outer coat.

To determine whether environmental conditions regulate the transcription of genes involved in crust synthesis during sporulation we performed qRT-PCR experiments. Four genes were targeted: *cotY*, *cgeA*, *spsI*, and *spsM*. *spsI* is the first gene of the *spsIJKL* locus that encode the enzymes of a pathway converting D-glucose-1-phosphate to dTDP-L-rhamnose. The *spsM* gene encodes the first enzyme of the pathway that converts UDP-GlcNAc to Leg and it was shown that this pathway is required for proper crust synthesis and/or anchoring (Dubois et al., 2020). In the *B. subtilis* 168 strain, the *spsM* gene is interrupted by the prophage SPβ. Excision of SPβ occurs in the mother cell of sporulating cells during the middle-to-late stages of sporulation, thus reconstituting a functional *spsM* gene (Abe et al., 2014, 2017). Therefore, any change in prophage excision potentially causes a change in crust synthesis and spore surface properties. Depletion in calcium or magnesium during sporulation did not impact the transcription of the targeted genes indicating that these sporulation conditions prevent crust assembly or negatively regulate crust synthesis at a post-transcriptional level. Post-transcriptional regulation of crust synthesis by divalent cations remains unknown but it might be mediated by glycosyltransferases that participate in the assembly of crust glycans. Indeed, nine glycosyltransferases are potentially involved in the synthesis and/or the anchoring of crust glycans on the spore surface: SpsA, SpsB, YfnF, YfnE, YfnD, YtcC, CgeD, CgeB and CotSA (Shuster et al., 2019a,b; Dubois et al., 2020). At least three of them (SpsA, YfnE, and CgeD) are putative GT-A type glycosyltransferases, a class of enzymes that are dependent on divalent metal ions. The metal ion is coordinated by a highly conserved DXD motif of the active site and it aids leaving group departure by stabilizing the charged phosphate groups in the nucleotide sugar donor (Gloster, 2014). Therefore, a depletion in divalent cations during sporulation might inhibit the activity of the GT-A glycosyltransferases and ultimately the assembly of glycans on the spore surface. When sporulation occurs in oxygen-limited conditions, transcription of genes involved in crust protein and glycans synthesis is decreased indicating that a lower availability in oxygen during sporulation represses crust synthesis at a transcriptional level. Transcription of the *spsM* gene was decreased in the -O_2_ and -O_2_ + CO_2_ conditions and transcription of *spsI* was also decreased in the -O_2_ + CO_2_ condition. Therefore, it is likely that the decrease in the relative amount of Leg and Rha in the crust of spores obtained in the oxygen-limited conditions is a consequence of the downregulation of the *sps* genes transcription during sporulation. Similarly, the decreased transcription of the *cotY* and/or *cgeA* genes might also explain the decrease in the relative amount of CotY and/or CgeA in the crust of spores obtained in oxygen-limited conditions. In contrast, the acidic pH did not modify transcription of the *cotY*, *cgeA*, *spsI*, and *spsM* genes suggesting that an acidic pH disrupts crust synthesis or assembly at a post-transcriptional level. It has been shown in *Bacillus* that acid stresses increase the intracellular amount of reactive oxygen species inducing secondary oxidative stress (Mols and Abee, 2011; Desriac et al., 2013). Therefore, the physiological changes and stress responses caused by acidic pH to sporulating cells may be similar to those induced by oxidizing molecules. It is well documented that oxidizing molecules can have a devastating effect on the structure and activity of proteins, especially those rich in sulfur amino acids (Ezraty et al., 2017). Moreover, it has been shown that the structural and morphogenetic proteins CotY and CotZ are rich in cysteines (9.3 and 6.7%, respectively) (Zhang et al., 1993). Therefore, an acidic pH during sporulation could induce a misfolding of CotY and/or CotZ, leading to the loss of their morphogenetic activity and ultimately to a defect in the crust proteins assembly. Transcription of all the genes targeted in qRT-PCR (*cotY*, *cgeA*, *spsI*, and *spsM*) is under the control of SigK and GerE and *cotY*, *cgeA*, and *spsI* genes transcription is also regulated by GerR (Figure 5A). Therefore, these transcriptional factors are potentially involved in the transcriptional regulation of these genes in response to sporulation conditions. Nevertheless, in the -O_2_ and -H_2_O_2_ conditions, only two out of the four genes were significantly less transcribed, suggesting a more complex regulation involving other regulators.

The crust is quite well conserved within the *Bacillus subtilis* group, although it is polymorphic from one species to another. For example, long flexuous filaments were observed on the surface of *B. licheniformis* spores, whereas the filaments were assembled into spikes on spores of *B. clausii* (Faille et al., 2014). However, the differences in crust composition and structure that explain these differences have not yet been identified. In clostridia and bacteria of the *B. cereus* group, the outermost layer of spores is the exosporium (Bozue et al., 2015; Stewart, 2015). Across species, the basic design of the exosporium consists of a thin continuous proteinaceous layer. In *B. cereus*, this basal layer is mainly composed of ExsY and CotY which are orthologs of CotZ and CotY in *B. subtilis*. The external face of the basal layer may be surrounded by a hairy nap made of collagen-like glycoproteins. In *B. cereus*, the main collagen-like glycoprotein is BclA (Sylvestre et al., 2002; Lequette et al., 2011). There is no collagen-like glycoprotein in the crust of *B. subtilis* suggesting major structural differences between crust and exosporium, particularly about glycans localization and anchoring. However, it seems that sporulation conditions also influence the structure of the exosporium of *B. cereus* spores. Indeed, when produced at high temperature or in anaerobic conditions *B. cereus* spores showed a damaged exosporium (Faille et al., 2007; Abbas et al., 2014). Therefore, interesting perspectives of this study would be to better characterize the diversity of the outermost layers of spores and further investigate the consequences of sporulation conditions on these layers, which are so crucial to the adhesion properties of spores.

To summarize, in *B. subtilis* a wide range of sporulation conditions can result in the production of spores with surface and adhesion properties very different from those produced under optimal conditions (Table 4). These sporulation conditions include divalent cations depletion, reduced oxygenation, oxidative stress, and acidic pH. Despite a similar effect on spore hydrophobicity and adhesion, the effect on the crust composition differs depending on the type of stress. Divalent cations depletion during sporulation results in a decrease in the amount of glycans on the spore surface and this decrease would be due to a crust assembly defect or a post-transcriptional regulation of glycan synthesis. By contrast, sporulation in oxygen-limited condition causes a decrease in the amount of glycans and proteins in the crust. This decrease is probably due to a repression of the transcription of genes involved in the synthesis of these compounds during sporulation. On the other hand, there is no clear trend in the consequences of oxidative stress and acidic pH during sporulation on the composition of the crust. Nevertheless, our results suggest that these categories of stresses affect the composition and/or structure of crust proteins rather than glycans.

The fact that sporulation conditions affect the ease with which spores can contaminate surfaces is therefore probably a major consideration in estimating the risk posed by *B. subtilis* spores in real environments. It could explain the frequent and recurrent presence of *B. subtilis* spores in food processing lines, despite laboratory results suggesting their low ability to contaminate surfaces. In view of these results, it could also be interesting to investigate the influence of environmental conditions encountered in the food industry during processing or cleaning operations on the surface properties of mature spores and their ability to adhere to all types of materials.

## Data availability statement

The raw data supporting the conclusions of this article will be made available by the authors, without undue reservation.

## Author contributions

AH: investigation, methodology, data collection, analysis, and writing. CL: investigation, methodology, data collection, and analysis. FK: investigation, data collection, and analysis. CF: supervision and writing – review and editing. TD: project administration, funding acquisition, conceptualization, supervision, investigation, methodology, and writing–review and editing. All authors contributed to the article and approved the submitted version.

## Funding

This work was supported by the Agence Nationale de la Recherche (SOGLOSSI project; ANR-21-CE21-0012). The Hauts-de-France region provided a Ph.D. grant to AH.

## Conflict of interest

The authors declare that the research was conducted in the absence of any commercial or financial relationships that could be construed as a potential conflict of interest.

## Publisher’s note

All claims expressed in this article are solely those of the authors and do not necessarily represent those of their affiliated organizations, or those of the publisher, the editors and the reviewers. Any product that may be evaluated in this article, or claim that may be made by its manufacturer, is not guaranteed or endorsed by the publisher.
