# Supplementary material for: Sporulation conditions influence the surface and adhesion properties of Bacillus subtilis spores
Source: Front Microbiol. 2023 Sep 1;14:1219581. doi: 10.3389/fmicb.2023.1219581 (PMC10502511; doi:10.3389/fmicb.2023.1219581)
Supplement: Supplementary file 1 [file Data_Sheet_1.pdf]

Supplementary Material

Sporulation conditions influence the surface and adhesion properties of *Bacillus subtilis* spores

Audrey Hamiot[1], Christelle Lemy[1], Frederic Krzewinski[2], Christine Faille[1] and Thomas Dubois[1*]

[1]Univ. Lille, CNRS, INRAE, Centrale Lille, UMR 8207 - UMET - Unité Matériaux et Transformations, F-59000 Lille, France

[2]Univ. Lille, CNRS, UMR 8576 - UGSF - Unité de Glycobiologie Structurale et Fonctionnelle, F-59000 Lille, France

*** Corresponding author:** Thomas Dubois:
thomas.dubois@inrae.fr

1 Supplementary Figures and Tables

1.1 Supplementary Figures

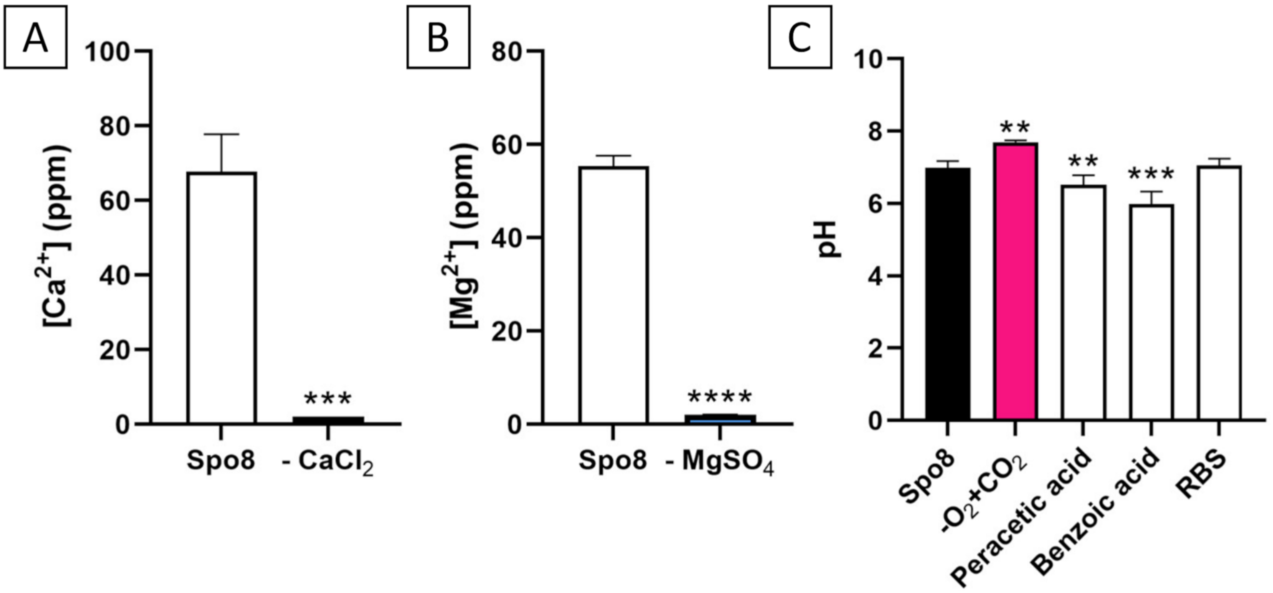

Supplementary Figure 1: Characterization of the chemical modifications resulting from changes in sporulation conditions. Dosage of (A) Ca^{2+} in the Spo8 and -$CaCl_2$ conditions and (B) Mg^{2+} in the Spo8 and -$MgSO_4$ conditions by photoelectric flame photometer. Concentrations are given in parts per million (ppm). Error bars represent the SDs of the means. ***, $p \leq 0.001$; ****, $p \leq 0.0001$ for - $CaCl_2$ or -$MgSO_4$ versus Spo8 by Welch's t-test. (C) pH of the medium in Spo8, -O_2+CO_2, peracetic acid, benzoic acid, and RBS conditions. Error bars represent the SDs of the means. **, $p \leq 0.01$; ***, $p \leq 0.001$ for each condition versus Spo8 by Mann-Whitney.

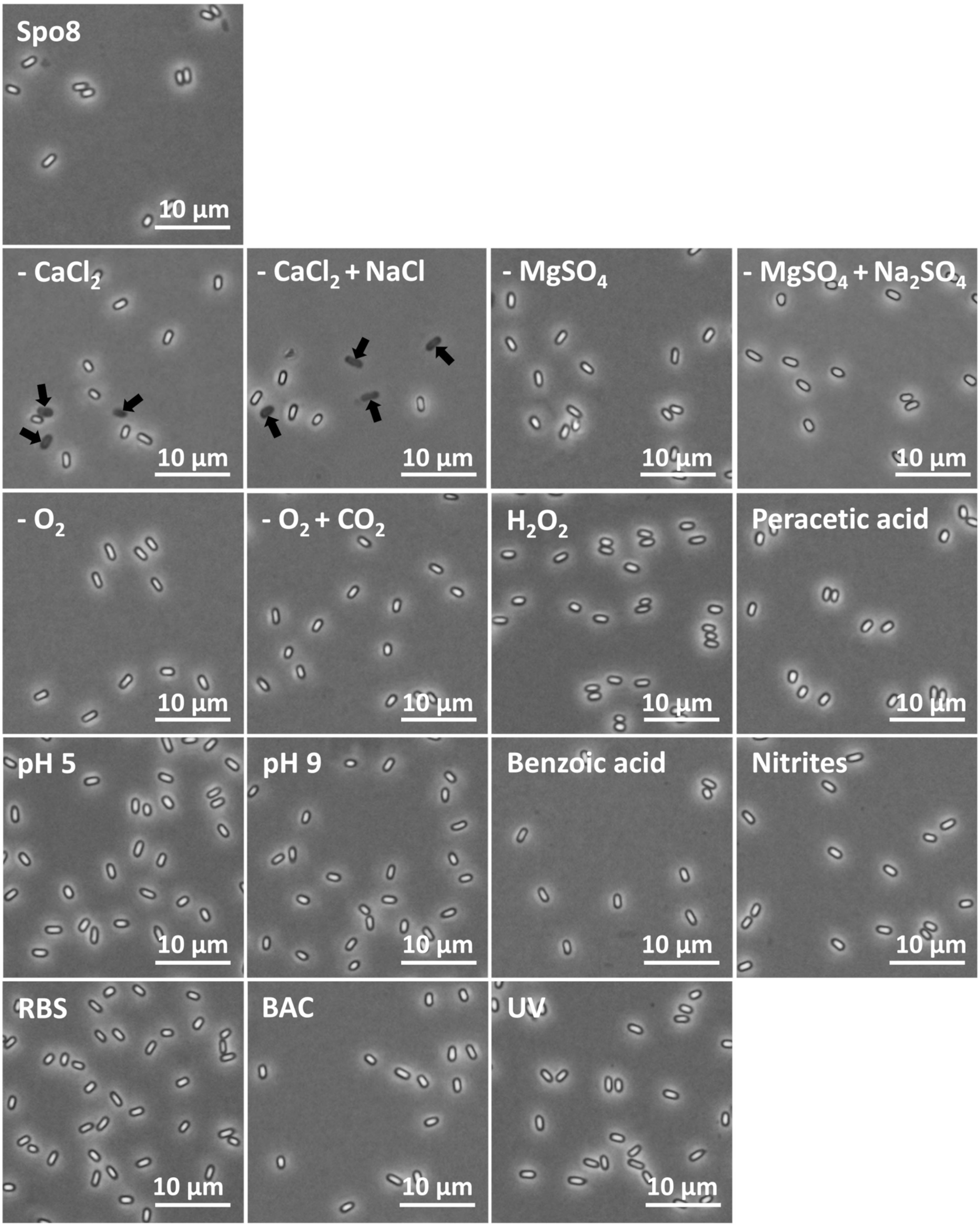

Supplementary Figure 2: Observation of *B. subtilis* spores by phase contrast microscopy. The black arrows point to non-refractive spores.

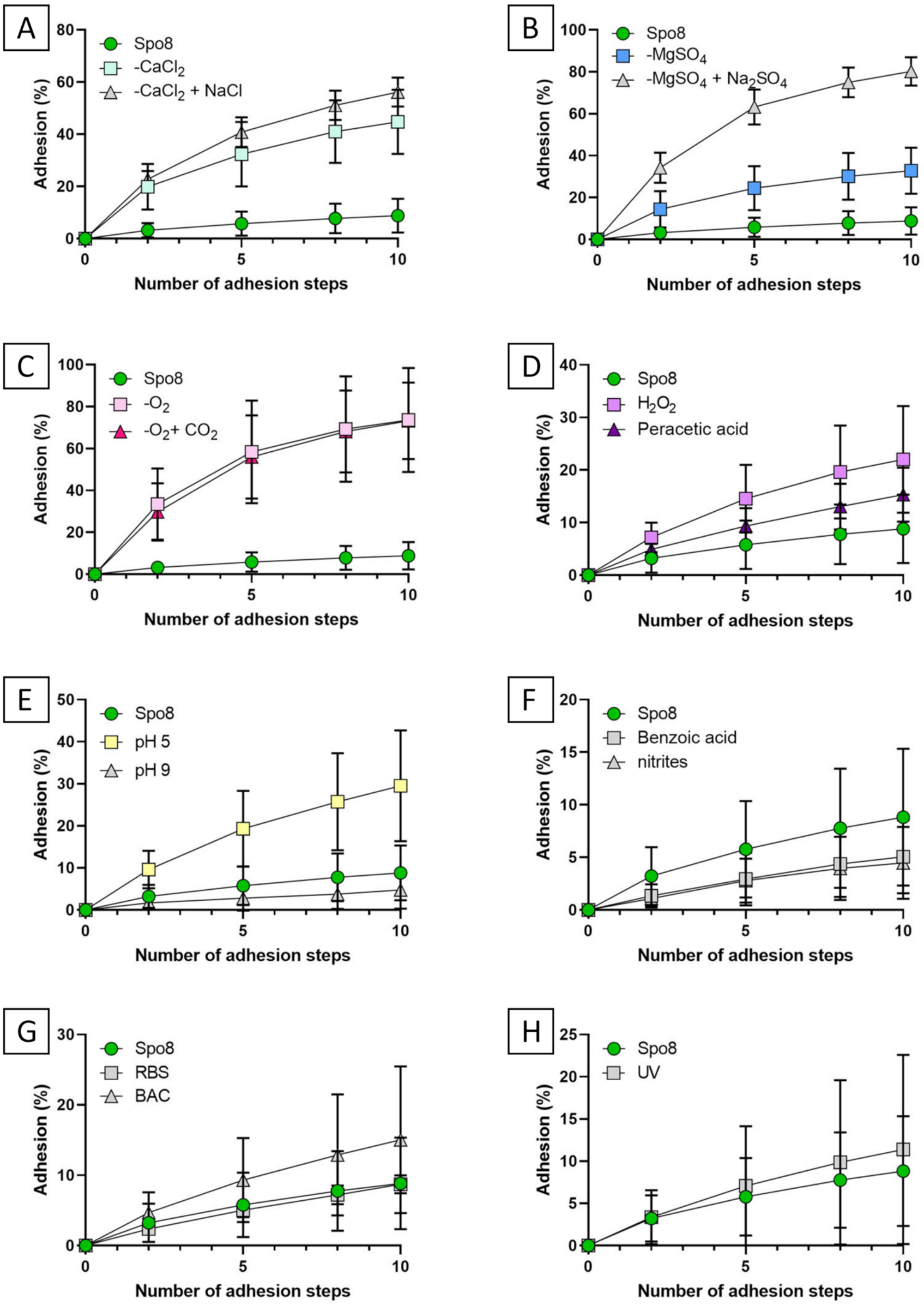

Supplementary Figure 3: Kinetics of spore adhesion to polypropylene. The adhesion of spores obtained after sporulation in Spo8, and the different sporulation conditions: -$CaCl_2$ or -$CaCl_2$ + NaCl (A), -$MgSO_4$ or -$MgSO_4$ + Na_2SO_4 (B), -O_2 or -O_2+CO_2 (C), H_2O_2 or peracetic acid (D), pH 5 or pH 9 (E), benzoic acid or nitrites (F), RBS or BAC (G), and UV (H) was evaluated after 0, 2, 5, 8 and 10 adhesion steps. Error bars represent the SDs of the means.

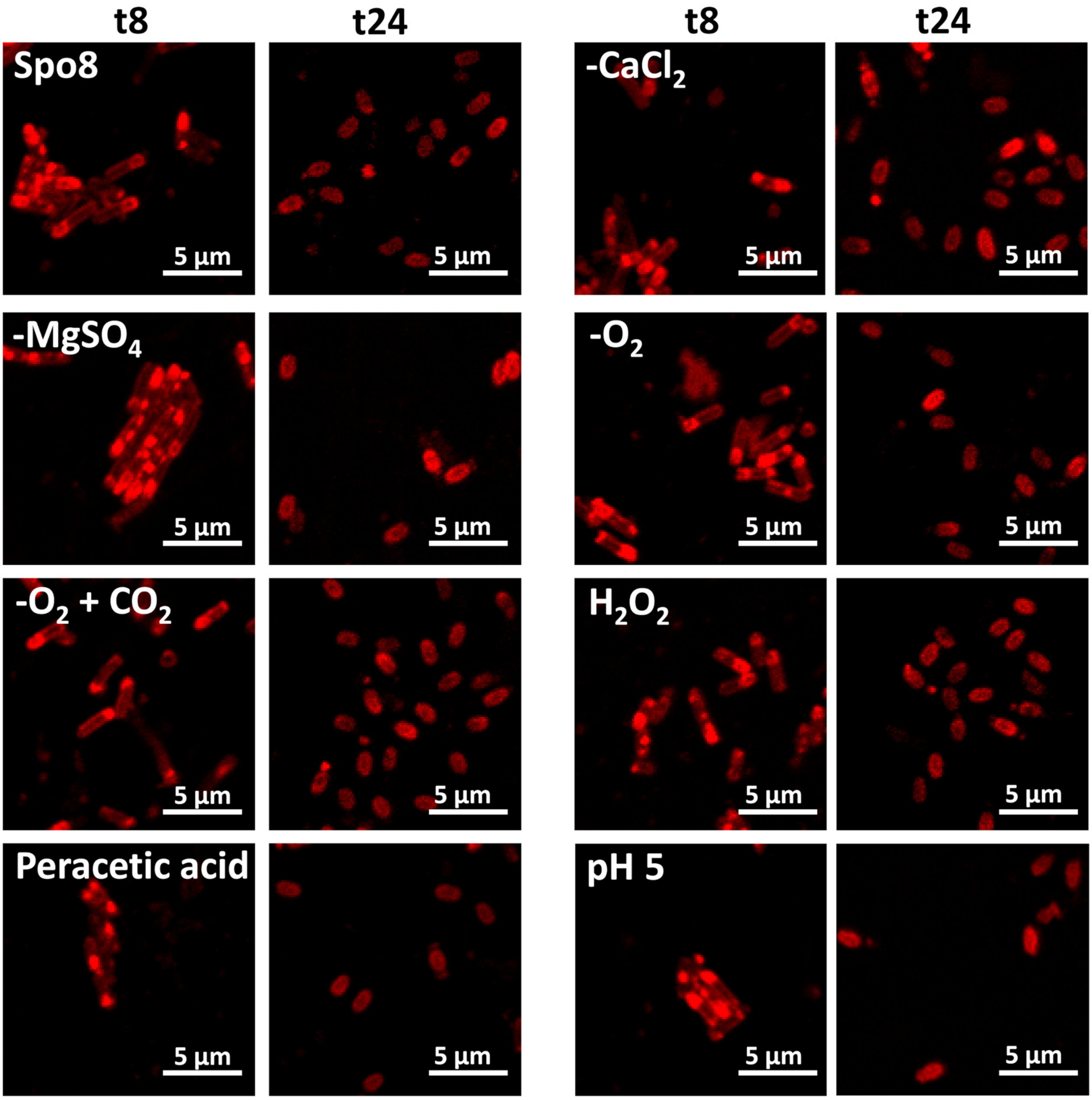

Supplementary Figure 4: Observation of *B. subtilis* during sporulation by confocal microscopy after FM4-64 staining. Sporulating cells were observed 8 hours and 24 hours after the transition to the stationary phase. FM 4-64 is a lipophilic dye staining membrane with red fluorescence.

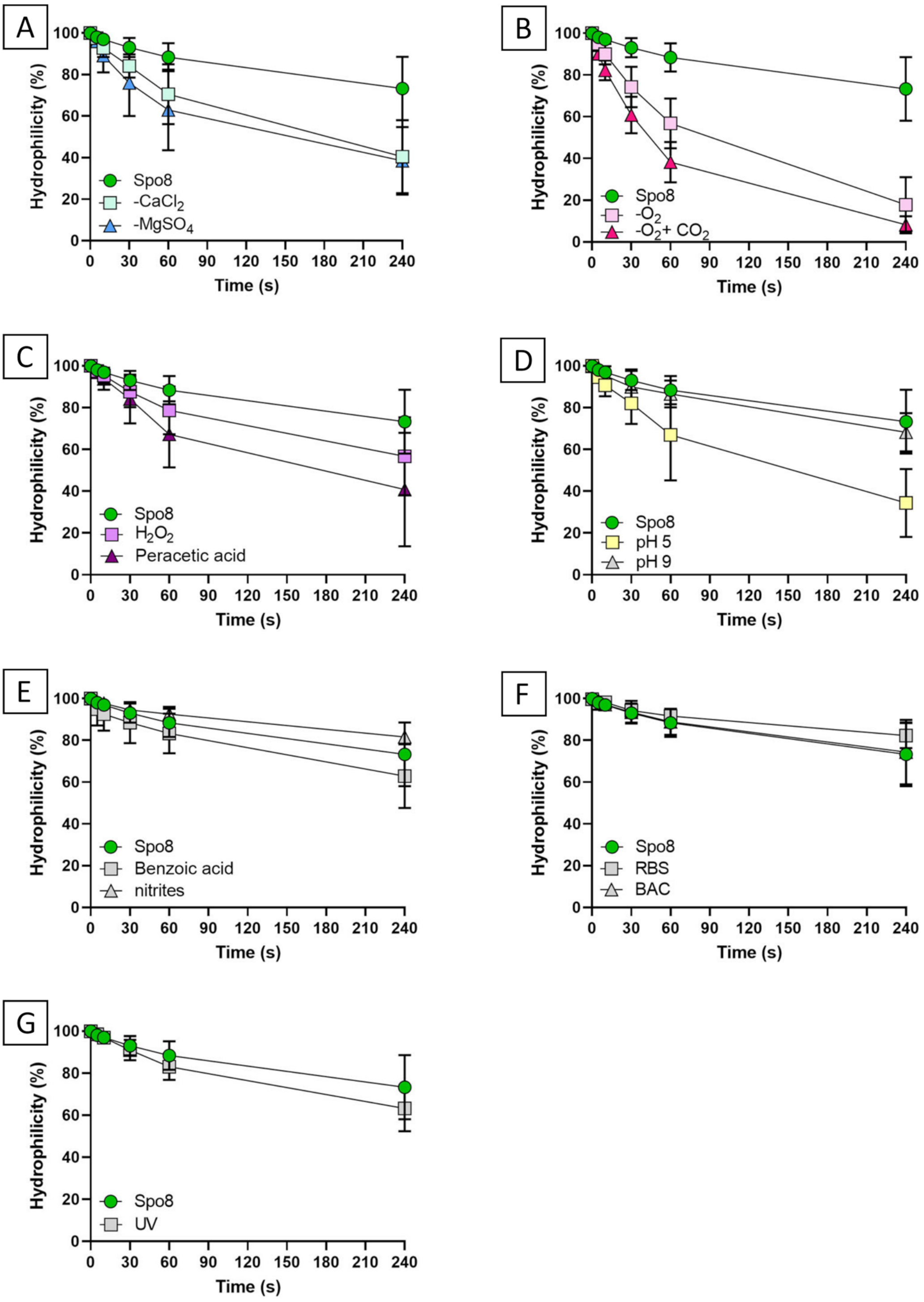

Supplementary Figure 5: Kinetics of MATH experiments. The percentage of hydrophilicity, given by the percentage of OD_{600} of the aqueous suspension before and after mixing is plotted against the vortexing time (s). Spores were produced in Spo8, and the different sporulation conditions: -$CaCl_2$ or -$MgSO_4$ (A), -O_2 or -O_2+CO_2 (B), H_2O_2 or peracetic acid (C), pH 5 or pH 9 (D), benzoic acid or nitrites (E), RBS or BAC (F), and UV (G). Error bars represent the SDs of the means.

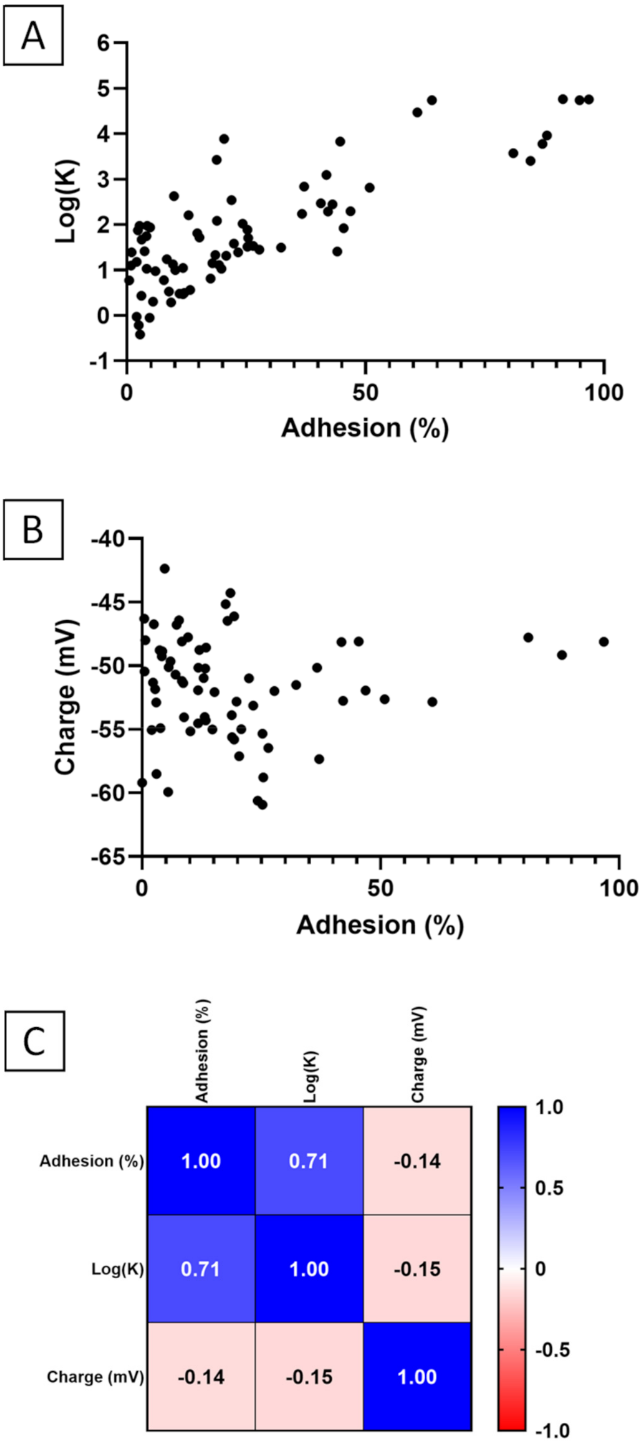

Supplementary Figure 6: Correlation between spore adhesion, hydrophobicity, and charge. (A) Scatter plot of the Log(K) as a function of the percentage of adhesion after ten binding reactions. (B) Scatter plot of the charge of spores as a function of the percentage of adhesion after ten binding reactions. (C) Correlation matrix. The correlation factors were calculated using the Spearman correlation method from the results obtained in the thirteen conditions tested and the control condition.

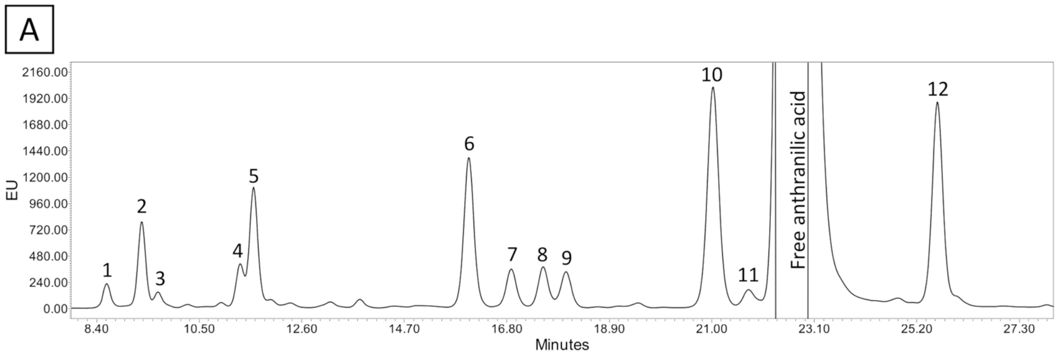
A
EU
Minutes
1
2
3
4
5
6
7
8
9
10
11
12
Free anthranilic acid

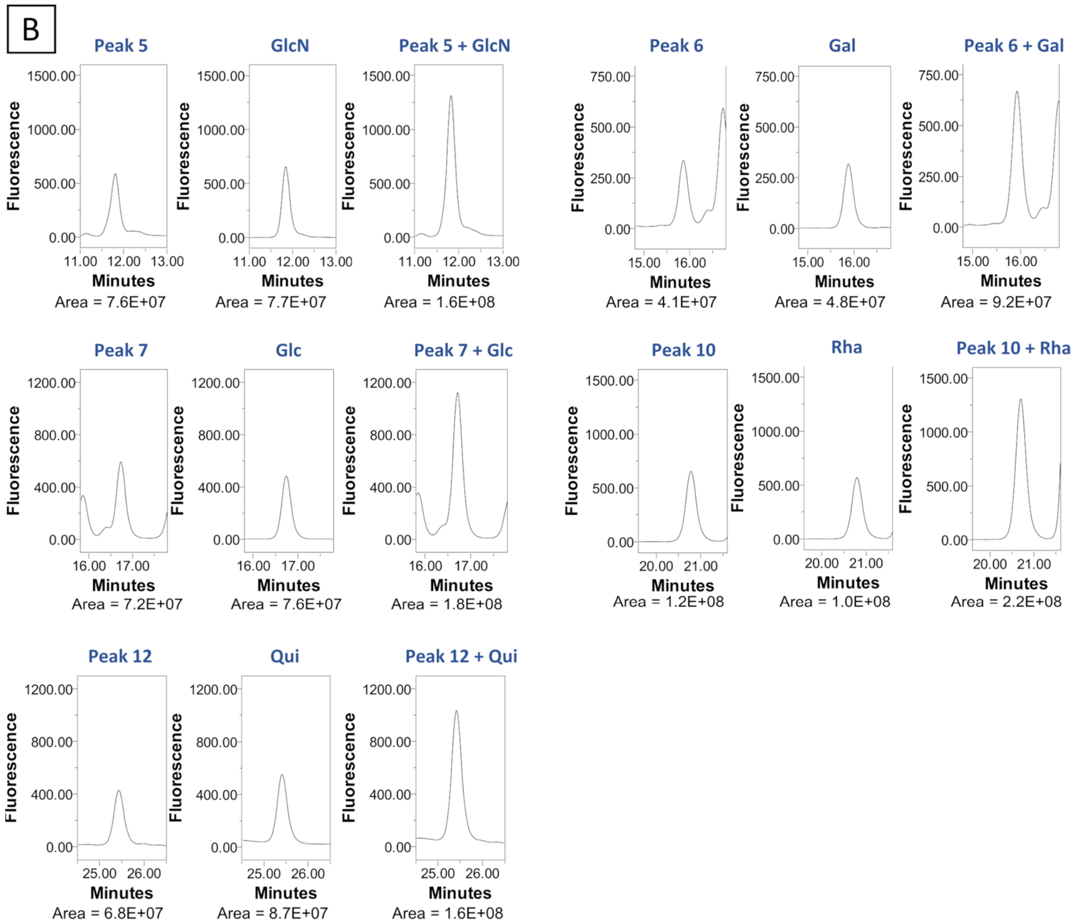
B
Peak 5
Area = 7.6E+07
GlcN
Area = 7.7E+07
Peak 5 + GlcN
Area = 1.6E+08
Peak 6
Area = 4.1E+07
Gal
Area = 4.8E+07
Peak 6 + Gal
Area = 9.2E+07
Peak 7
Area = 7.2E+07
Glc
Area = 7.6E+07
Peak 7 + Glc
Area = 1.8E+08
Peak 10
Area = 1.2E+08
Rha
Area = 1.0E+08
Peak 10 + Rha
Area = 2.2E+08
Peak 12
Area = 6.8E+07
Qui
Area = 8.7E+07
Peak 12 + Qui
Area = 1.6E+08
Fluorescence
Minutes

C

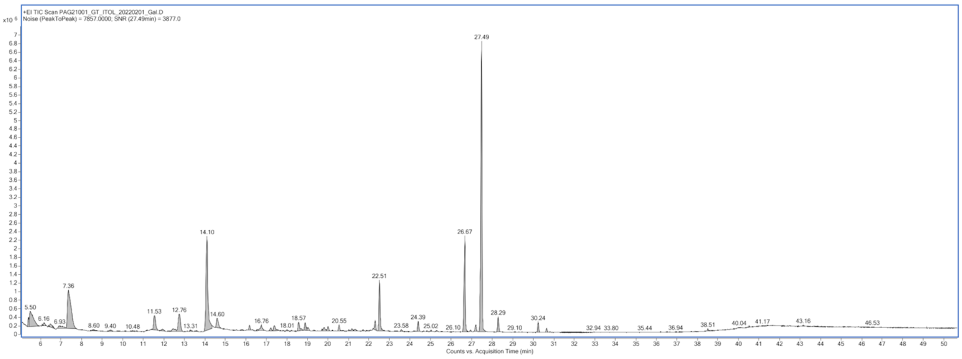
+EI TIC Scan PAG21001_GT_ITOL_20220201_Gal.D
Noise (PeakToPeak) = 7857.0000; SNR (27.49min) = 3877.0
x10 6
Counts vs. Acquisition Time (min)

D

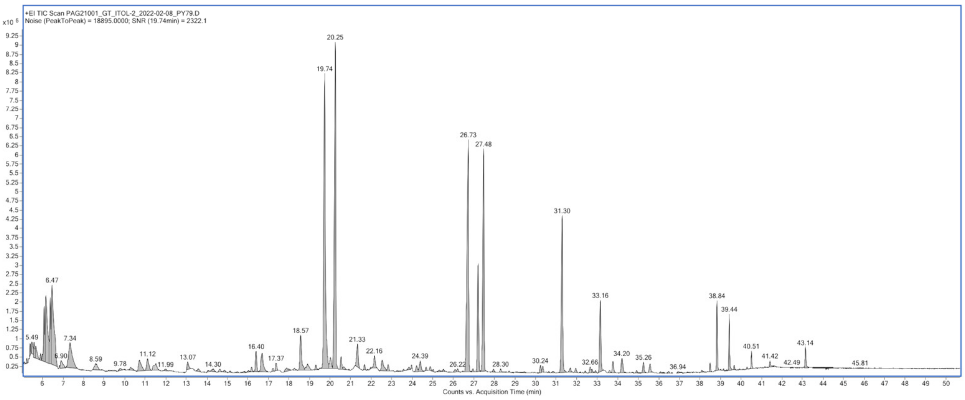
+EI TIC Scan PAG21001_GT_ITOL-2_2022-02-08_PY79.D
Noise (PeakToPeak) = 18895.0000; SNR (19.74min) = 2322.1
x10 6
Counts vs. Acquisition Time (min)

E

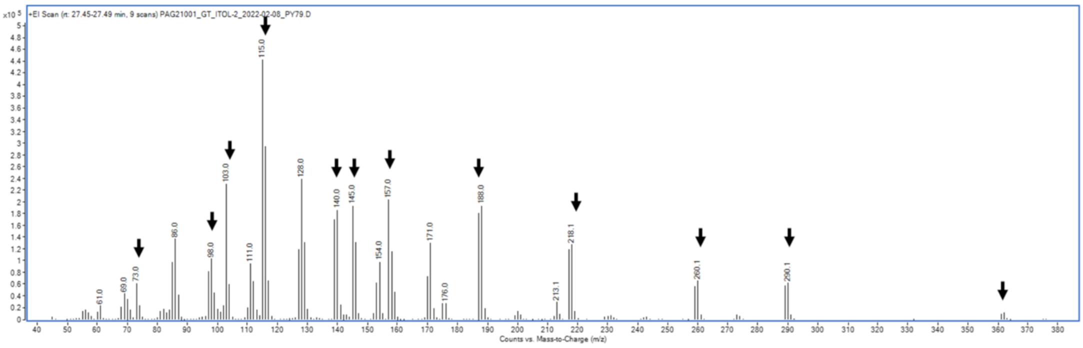

F

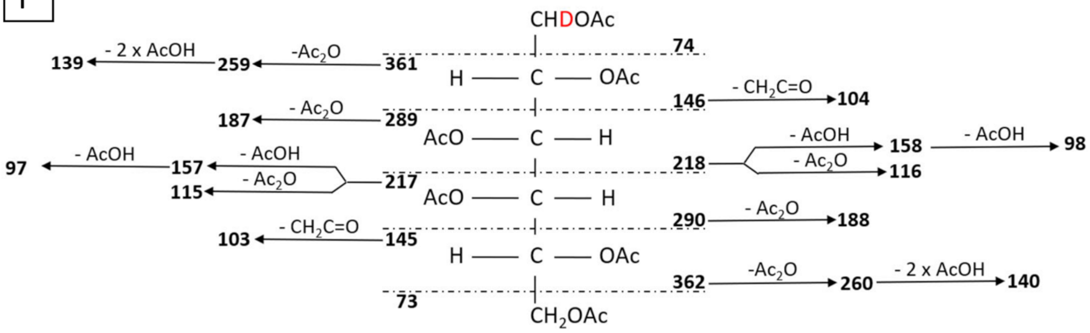

Supplementary Figure 7: Composition in monosaccharides of the crust of *B. subtilis* spores. (A) Chromatogram of the RP-HPLC-FL experiment performed on the crust of *B. subtilis* 168 spores obtained in the Spo8 condition after hydrolysis and derivatization by AA. Peaks corresponding to putative AA-sugars are numbered from 1 to 12. Peaks 1 to 3: putative disaccharides containing glycosidic linkages resistant to acid hydrolysis (Windwarder et al., 2016), peak 5: GlcN-AA, peak 6: Gal-AA, peak 7: Glc-AA, peak 10: Rha-AA, peak 12: Qui-AA. The molecules corresponding to peaks 4, 8, 9, and 11 are unknown. The large peak between 21.5 and 24 minutes corresponded to the excess of free anthranilic acid. (B) Chromatograms of RP-HPLC-FL experiments performed on a mix of monosaccharide standards and the crust of *B. subtilis* 168 spores. The retention times of peaks 5, 6, 7, 10, and 12 match those of GlcN-AA, Gal-AA, Glc-AA, Rha-AA, and Qui-AA standards, respectively (C) Chromatogram of the GC-MS experiments performed on hexa-acetate galactitol standard. (D) Chromatogram of the GC-MS experiments performed on the crust of *B. subtilis* 168 spores. (E) GC-MS spectra of hexa-acetate galactitol. The characteristics of ions are indicated by black arrows. (F) Structure of hexa-acetate galactitol and the prominent fragment ions. The reduction of sugars was performed with borodeuteride.

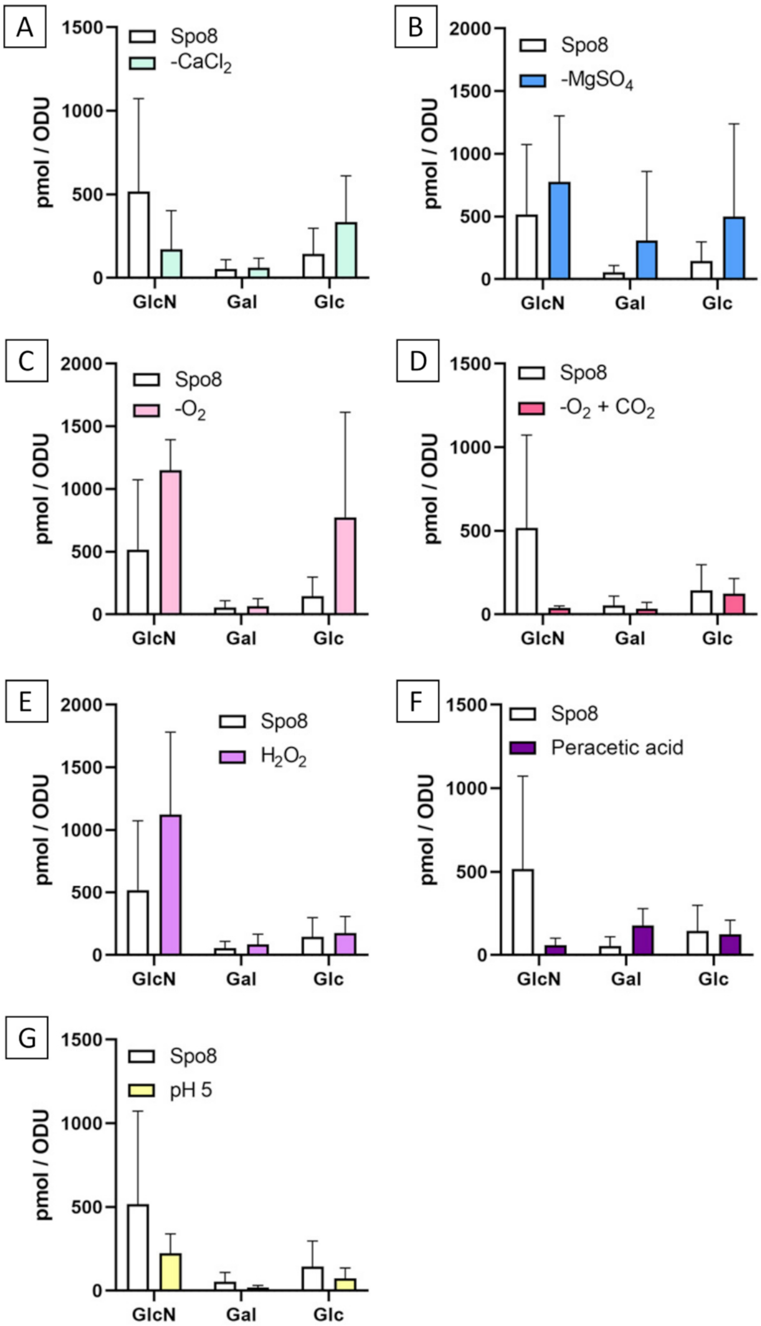

Supplementary Figure 8: Influence of sporulation conditions on the crust composition in monosaccharides. The relative amounts of GlcN, Gal, and Glc were evaluated by RP-HPLC-FL. The experiments were performed on the crust of spores released from *B. subtilis* 168 grown in Spo8, and in the sporulation conditions shown to affect spore surface properties: -$CaCl_2$ (A) -$MgSO_4$ (B), -O_2 (C), -O_2+CO_2 (D), H_2O_2 (E), peracetic acid (F) and pH 5 (G). The results were standardized by the OD_{600nm} of the spore preparations. Error bars represent the SDs of the means. *, $p \leq 0.05$; **, $p \leq 0.01$; ***, $p \leq 0.001$ for each condition versus Spo8 by Mann-Whitney.

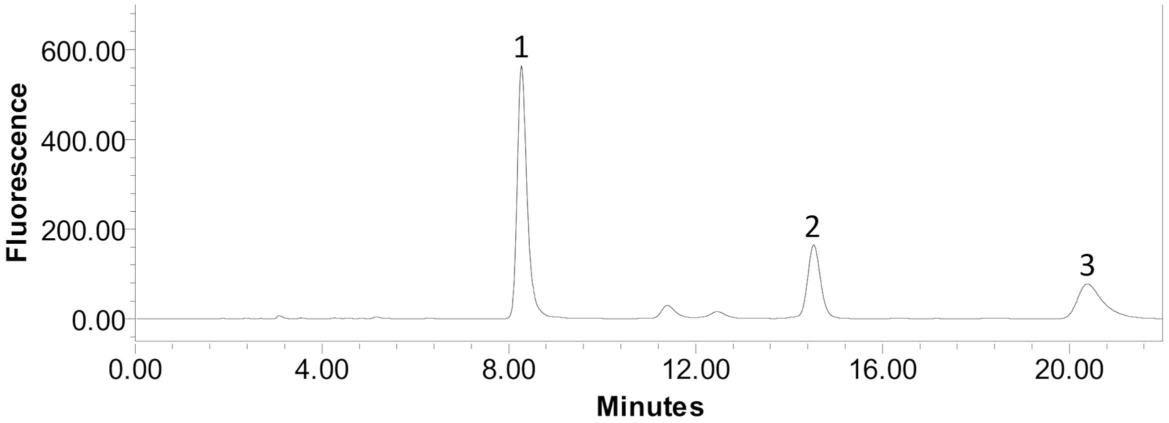

Supplementary Figure 9: Chromatogram of a RP-HPLC-FL experiment performed after hydrolysis and DMB derivation of the crust of *B. subtilis* 168 spores prepared in the Spo8 condition. Peak 1: DMB-Leg, Peaks 2 & 3: unknown molecules.

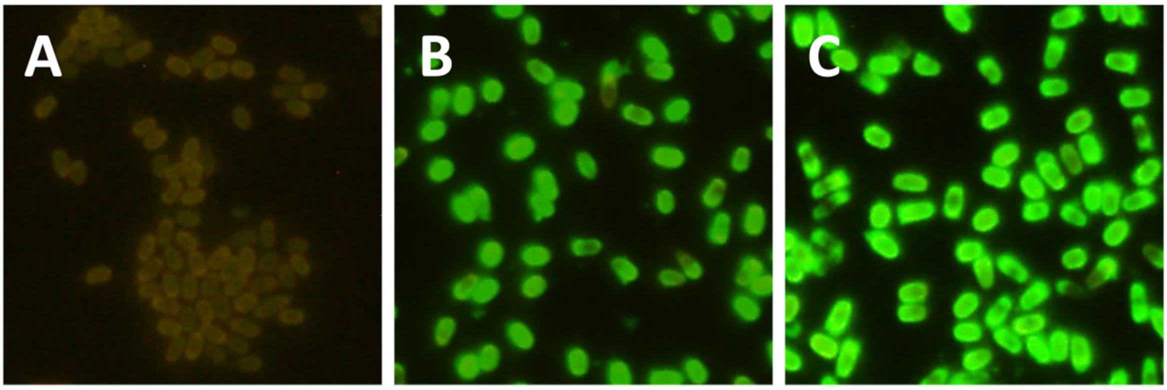

Supplementary Figure 10: Observation of spores by fluorescence microscopy. Spores of the *B. subtilis* 168 (A), *B. subtilis* 168 *amyE*::*cgeA*-GFP (B), and *B. subtilis* 168 *amyE*::*cotY*-GFP (C) obtained in the Spo8 condition were observed by fluorescence microscopy to evaluate fluorescence and localization of the CgeA-GFP and CotY-GFP fusion proteins. Spores of the *B. subtilis* 168 strain were used as a negative control to estimate the natural autofluorescence of *B. subtilis* at the excitation wavelength of GFP.

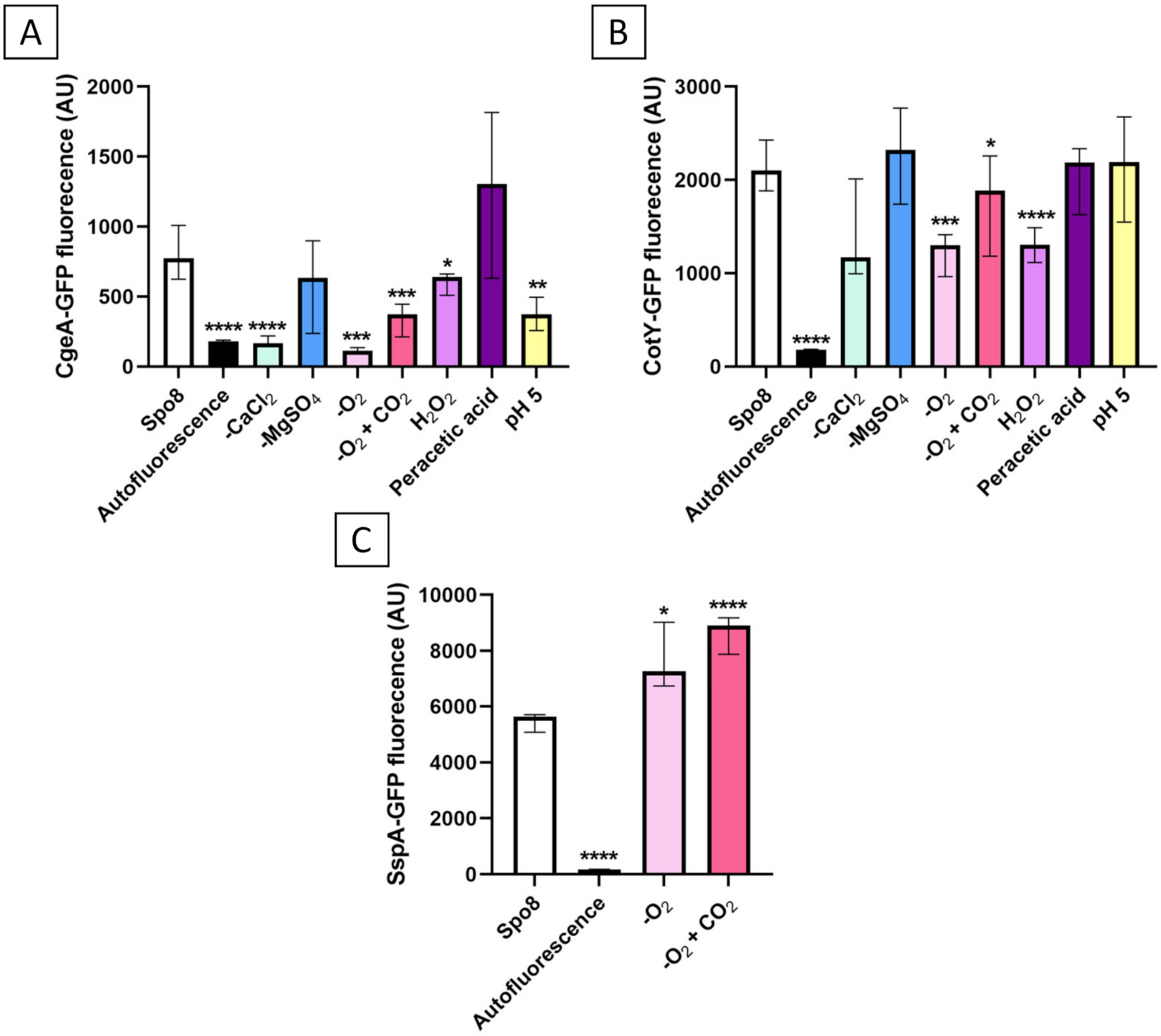

Supplementary Figure 11: Evaluation of the relative amount of CgeA-GFP and CotY-GFP on the spore surface by flow-cytometry experiments. Fluorescence of the CgeA-GFP (A) or CotY-GFP (B) fusion proteins on the spore surface was measured by flow cytometry after sporulation of the B. subtilis 168 *amyE*::*cotY*-GFP or *B. subtilis* 168 *amyE*::*cgeA*-GFP strain in the Spo8, and in the sporulation conditions shown to affect spore surface properties: -$CaCl_2$, -$MgSO_4$, -O_2, -O_2+CO_2, H_2O_2, peracetic acid and pH 5. Autofluorescence: Fluorescence of spores of the *B. subtilis* 168 strain. (C) Control of the GFP fluorescence in the -O_2 or -O_2+CO_2 condition. Fluorescence of the SspA-GFP fusion protein in the core of spores was measured by flow cytometry after sporulation of the *B. subtilis* 168 pHT304-18 P_{sspA}-*sspA*-GFP in the Spo8, -O_2 and -O_2+CO_2 conditions. SspA: small acid-soluble spore protein. This control was performed to ensure that GFP fluorescence is not decreased under conditions of lower oxygenation during sporulation. Indeed, GFP requires molecular oxygen for the maturation of fluorescence. Error bars represent the median with the interquartile range. *, $p \leq 0.05$; **, $p \leq 0.01$; ***, $p \leq 0.001$; ****, $p \leq 0.0001$ for each condition versus Spo8 by t-test, Welch's t-test, or Mann-Whitney, depending on the result of the normality test and F-test.

1.2 Supplementary Tables

Supplementary Table 1: Chemicals used for this study.

Chemical	Supplier	Reference number
Sodium bicarbonate	Acros Organics	123360010
H_2O_2	Sigma-Aldrich	H1009
Peracetic acid	Acros Organics	257751000
HCl	PanReac	211020.1611
NaOH	Fisher Chemical	S/4950/PB15
Benzoic acid	Acros Organics	423475000
Nitrites	Fisher Chemical	S/5600/53
RBS	RBS	T105
Benzalkonium Chloride	Acros Organics	215411000

Supplementary Table 2: Primers used for this study. Restriction sites are in bold and underlined. Overlapping sequences used for SOE PCRs are in bold and colored in green, blue, or orange. Sequences in green encode linkers consisting of five glycines that were introduced between CgeA or CotY and GFP.

Primer	Sequence	Restriction site
**gfp-1**	**GGAGGCGGAGGTGGA**AGTAAAGGAGAAGAACTTTTCACTG	
**gfp-2**	**GATGCCTCAAGCTAGAGAGTCACGGCCGCTACTAGTATTTGTAG**	
**λter-1**	**CTACAAATACTAGTAGCGGCCGTGACTCTCTAGCTTGAGGCATC**	
**λter-2**	AA**<u>CTGCAG</u>**GTTAACAAGAGTTTGTAGAAACGC	*Pst*I
**cgeA-1**	GC**<u>TCTAGA</u>**ATCATCCCTCTCCCTTATCCT	*Xba*I
**cgeA-2**	**TCCACCTCCGCCTCC**TGAAAAGAACGTAACGCTTTCT	
**cotY-1**	GC**<u>TCTAGA</u>**ACGTCACAACAGTAGATGCC	*Xba*I
**cotY-2**	**TCCACCTCCGCCTCC**TCCATTGTGATGATGCTTTTTATC	
**polC-F**	AGGACTTGATGCTTCACCGA	
**polC-R**	CTCTCACACATCTCAAGCGC	
**spsM-F**	AGGACCATCCATTTGAAGCG	
**spsM-R**	ACATTTTGTCCGCCGATGAG	
**SPβ-R**	GACGTGGCGGTTTGCTTGA	
**SPβ-F**	CACACCGAGTGCAGCATGT	
**cgeA-F**	GCCTTGCTTGATGCTGCCAT	
**cgeA-R**	GGCGTAATTCTGGCAAAAATCC	
**cotY-F**	GTCCGACTGGCTGCTATAC	
**cotY-R**	CCCTACGTTTCCGAATGTGG	
**spsI-F**	GCCTTATACAGAACGCTTTGAAC	
**spsI-R**	CTTGTTTTTCTCATCAATCTCAGCA	

Supplementary Table 3: Influence of sporulation conditions on the number of CFU, the percentage of heat-resistant spores, and the germination rate. The data presented in the table are the means with SDs. *, $p \leq 0.05$; **, $p \leq 0.01$; ***, $p \leq 0.001$; ****, $p \leq 0.0001$ for each condition versus Spo8 by t-test, Welch's t-test, or Mann-Whitney, depending on the result of the normality test and F-test.

Sporulation condition	N CFU / mL before heating ($x10^8$)	Heat-resistant spores (%)	Germination (%)
Spo8	6.38 ± 1.62	88.1 ± 11.1	46.8 ± 5.7
-$CaCl_2$	2.22 ± 0.30****	82.9 ± 8.8	28.0 ± 6.6****
-$MgSO_4$	7.24 ± 2.40	94.9 ± 4.8	48.1 ± 3.6
-O_2	10.1±2.16****	96 ± 6.4	39.0 ± 11.7
-O_2+CO_2	8.64 ± 0.48**	84.7 ± 14.6	47.6 ± 4.1
H_2O_2	6.13 ± 1.53	86.4 ± 13.4	44.1 ± 9.1
Peracetic Acid	7.91 ± 2.51*	84.7 ± 14	47.0 ± 1.7
pH 5	8.24 ± 1.86***	87.6 ± 12.1	45.4 ± 8.7
pH 9	7.16 ± 2.28	95.1 ± 6.3	43.0 ± 6.5
Benzoic Acid	6.51 ± 1.50	88.4 ± 4.7	41.2 ± 7.2
Nitrites	6.05 ± 2.74	90.9 ± 8.8	38.1 ± 11.7
RBS	6.47 ± 3.25	86.0 ± 17.0	41.5 ± 1.2
BAC	7.68 ± 2.66	85.32 ± 10.9	40.1 ± 9.6
UV	5.99 ± 0.79	82.1 ± 11.9	41.7 ± 9.5

Reference

Windwarder, M., Figl, R., Svehla, E., Mócsai, R. T., Farcet, J. B., Staudacher, E., et al. (2016). "Hypermethylation" of anthranilic acid-labeled sugars confers the selectivity required for liquid chromatography-mass spectrometry. *Anal. Biochem.* 514, 24-31. doi: 10.1016/j.ab.2016.09.008.
